# Dopamine-modified TiO_2_ monolith-assisted LDI MS imaging for simultaneous localization of small metabolites and lipids in mouse brain tissue with enhanced detection selectivity and sensitivity†
†Electronic supplementary information (ESI) available. See DOI: 10.1039/c7sc00937b



**DOI:** 10.1039/c7sc00937b

**Published:** 2017-03-21

**Authors:** Qian Wu, James L. Chu, Stanislav S. Rubakhin, Martha U. Gillette, Jonathan V. Sweedler

**Affiliations:** a Department of Chemistry , University of Illinois at Urbana-Champaign , 600 S. Mathews Ave, 63-5 , Urbana , Illinois 61801 , USA . Email: jsweedle@illinois.edu; b Department of Cell and Developmental Biology , University of Illinois at Urbana-Champaign , Urbana , Illinois 61801 , USA; c Beckman Institute , University of Illinois at Urbana-Champaign , 405 N. Mathews Ave, 63-5 , Urbana , Illinois 61801 , USA

## Abstract

Localization of metabolites using multiplexed mass spectrometry imaging (MSI) provides important chemical information for biological research. In contrast to matrix-assisted laser desorption/ionization (MALDI), TiO_2_-assisted laser desorption/ionization (LDI) for MSI improves detection of low molecular mass metabolites (<500 Da) by reducing matrix background. However, the low UV absorption of TiO_2_ nanoparticles and their ester hydrolysis catalytic activity hinder the detection of phospholipids and many low-abundance molecules. To address these challenges, we evaluated and optimized the material morphology and composition of TiO_2_. Dopamine (DA) was found to be an efficient ligand for TiO_2_, resulting in increased UV light absorption, higher surface pH, and formation of monolithic TiO_2_-DA structures. The sub-micron scale and higher surface pH of the TiO_2_ particle sizes led to improved detection of phospholipid signals. Compared to unmodified TiO_2_ sub-micron particles, the DA-modified TiO_2_ monolith led to 10- to 30-fold increases in the signal-to-noise ratios of a number of compound peaks. The TiO_2_-DA monolith-assisted LDI MSI approach has higher selectivity and sensitivity for Lewis basic compounds, such as fatty acids, cholesterols, ceramides, diacylglycerols, and phosphatidylethanolamine, when analyzed in positive mode, than traditional MALDI MS. Using this new method, over 100 molecules, including amino acids, alkaloids, free fatty acids, peptides, and lipids, were localized in mouse brain sections. By comparing the presence and localization of those molecules in young and old mouse brains, the approach demonstrated good performance in the determination of aging-related neurochemical changes in the brain.

## Abstract 2

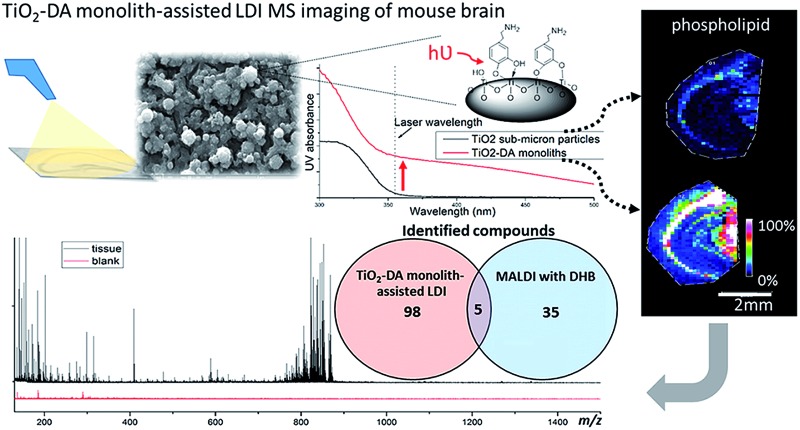
A dopamine-modified TiO_2_ monolith was developed to assist LDI MS imaging for small metabolites and lipids simultaneously with enhanced sensitivity.

## Introduction

Mass spectrometry imaging (MSI),^1^ a sensitive and multiplexed approach for the characterization and localization of a wide range of analytes, is used for both targeted and untargeted analyses. The field encompasses a variety of desorption/ionization techniques, including secondary ion mass spectrometry imaging, laser desorption/ionization (LDI) MSI, matrix-assisted laser desorption/ionization (MALDI) MSI,^2^ and desorption electrospray ionization MSI. Of these, MALDI MSI enables analyses across the widest molecular mass range^3–6^ at micrometer-scale spatial resolution, with analyte detection limits comparable to other MS-based approaches.^7,8^ MALDI MSI is well suited for the detection and localization of small metabolites,^4,6^ lipids,^9^ peptides, and proteins^10^ in tissues. The method can be used to reveal molecular mechanisms of disease,^11^ facilitate biomarker discovery,^12^ and unveil the chemical mechanisms of different physiological processes, such as cell-to-cell signaling,^13^ cell metabolism,^14^ and brain aging.^15^

Traditional MALDI matrixes, such as 2,5-dihydroxybenzoic acid (DHB), α-cyano-4-hydroxycinnamic acid, and 9-aminoacridine (9-AA), are small molecules with high UV light absorption and good desorption/ionization abilities.^16^ These widely used matrixes are indispensable in bioanalytical measurements of lipids, peptides, and proteins. However, due to the presence of multiple, intense MALDI matrix-related signals in the resulting mass spectra, their use in the detection of endogenous small molecules is limited.^17^ As one means to address this challenge, on-tissue derivatization was developed to increase the molecular mass of small analytes and assist targeted analysis.^18^ Other limitations of MALDI have also been reported, including ion suppression of targeted analytes by compounds that exhibit more efficient ionization and/or are present at high abundance,^16,19^ leading to lower selectivity.^20^ To overcome these drawbacks, nanomaterials such as porous silicon,^14^ carbon nanomaterials,^21–23^ gold/silver nanoparticles,^24^ and metal oxide nanoparticles^17^ have been tested as alternatives to traditional MALDI matrixes and used in approaches that are commonly referred to as surface- or nanoparticle-assisted laser desorption/ionization (LDI).^25,26^ In contrast to MALDI, the distinct ionization mechanism of nanoparticle-assisted LDI results in fewer background signals. Also, choosing a nanomaterial with an appropriate affinity for a specific analyte may increase the selectivity of the mass spectrometry (MS) analysis. Moreover, the deposition of thin layers of nanoscale-sized particles onto a biological sample results in a uniform coating, which facilitates high spatial resolution in MSI.

Among the available nanomaterials, TiO_2_ has been widely used to assist LDI.^27^ Compared with silicon and gold or silver nanoparticles, which are not always stable in an ambient environment (*e.g.*, surface oxidation) or under laser irradiation (resulting in the generation of artifacts such as Au^*n*^ clusters),^24,27^ TiO_2_ creates a stable layer on samples. Carbon nanomaterials for LDI also offer great potential, although they are reported to be hard to disperse in solution^28^ and can be dislodged, resulting in contamination of an instrument's ion source.^21^ TiO_2_-assisted LDI MS has been used in the detection of r-cyclodextrin,^29^ steroid hormones,^28,30^ and peptides and small proteins,^31^ as well as other neutral small molecules such as catechins.^32^ TiO_2_-assisted LDI MSI was successfully applied to localize small molecules (molecular mass below 500 Da) in the mouse brain;^33^ however, few endogenous lipids, which are critical components in biological systems, were detected. The detection of several phosphatidylcholines (PCs) and triacylglycerides with TiO_2_ particles in soy bean extracts has been reported.^34^ The authors also proposed that TiO_2_ acts as a catalyst for phospholipid hydrolysis, and interestingly, the use of TiO_2_ microparticles instead of TiO_2_ nanoparticles resulted in relatively higher signals for lipids. In recent MSI studies using TiO_2_ nanoparticles, the degradation of esters was also found.^35^

Several studies have demonstrated that modifications to TiO_2_ can impact its catalytic and other physiochemical properties. Some specific nanostructured TiO_2_ methods provide faster hydrolysis of organophosphorus esters.^36^ The Lewis acid site in TiO_2_ (Ti^4+^) has a strong affinity to Lewis bases, such as phosphoric salt,^37^ or organic anchoring groups, including *cis*-enediol compounds, phosphonate, and hydroxyl carboxylic acid;^38–40^ the coordination of Ti with these ligands can cause a red shift to its UV absorption. Recently, modified TiO_2_ particles have been used in TiO_2_-assisted LDI analysis^30,32^ for targeted analyte detection, including steroid hormone measurements.

Since it is important to characterize larger lipids^41–44^ and small metabolites,^45–49^ many of which are hard to characterize using existing methods, we modified the surface properties of TiO_2_ to change its catalytic activity and increase its UV absorption, and then evaluated the modifications to the structure of TiO_2_ using different Lewis base ligands. Dopamine (DA), the optimal ligand, was found to largely increase the UV absorption of TiO_2_, leading to an increase in the sensitivity of MSI detection. The micro-morphology of TiO_2_ was also optimized, allowing formation of monolithic structures that led to an increase in active surface area. In addition, the pH environment on the TiO_2_ monolithic surface was increased, reducing the catalytic hydrolysis of the lipids. Using our approach, over 50 lipids were detected in the mouse brain, together with 35 metabolites with molecular weights under 500 Da. We validated the method using the aging brain model, in which many compounds, including proteins,^42^ lipids,^41–44,48^ and small metabolites,^45–49^ have been reported to change during aging, but as of yet have not been localized together. Our imaging results combined with principal component analysis (PCA) revealed important chemical differences between old and young animals, including the determination of six molecules with signal levels that changed in an age-dependent manner. These results offer a new perspective on the chemical signature of brain aging.

## Experimental section

### Chemicals and materials

The following chemicals were purchased from Sigma-Aldrich (St. Louis, MO): titanium(iv) *n*-butoxide, dopamine hydrochloride (>98%), alizarin (>97%), ascorbic acid (>98%), salicylic acid (SA) (>99%), DHB, phospholipid mixture (1 mg mL^–1^, HPLC grade), cholesterol (>99%), *N*-hexanoyl-d-sphingosine (>98%), galactocerebrosides mixture (1 mg mL^–1^, HPLC grade), linolenic acid (purity ≥ 99%), linoleic acid (purity ≥ 99%), erucic acid (purity ≥ 99%), elaidic acid (purity ≥ 99%), and palmitoleic acid (purity ≥ 98.5%). Concentrated nitric acid and phosphoric acid (analytical grade), acetonitrile, ethanol, and water (liquid chromatography (LC)/MS grade) were purchased from Fisher Scientific (Pittsburgh, PA).

### Synthesis of TiO_2_ nanoparticles, sub-micron particles, and monolith

TiO_2_ nanoparticles were prepared using a previously described sol–gel method to hydrolyze titanium(iv) *n*-butoxide in an ethanol–water solution under acidic conditions.^33^ Briefly, 3.4 mL of titanium(iv) *n*-butoxide and 1.6 mL of ethanol were mixed by vortex for 1 min, forming a precursor solution. Then, a solution containing 5 mL of ethanol with 0.1 M nitric acid and 1% HPLC grade water was added dropwise to the vigorously stirred precursor solution, which was cooled in an ice/water bath. TiO_2_ structures were formed in this solution (solution I) during stirring in an ice/water bath for 3 h.

In preparation of the TiO_2_ nanoparticle deposition mixture, 250 μL of solution I were directly diluted in 5 mL ethanol. Modified TiO_2_ nanoparticle deposition mixtures were prepared by the addition of 0.005 M ligands (DA, alizarin, ascorbic acid, or SA) into the diluted solution I followed by a 30 min incubation. In preparation of the TiO_2_ sub-micron particle deposition mixture and DA-modified TiO_2_ monolith deposition mixture, 250 μL of solution I were diluted in 5 mL of reaction solution for further hydrolysis/condensation (solution II). For TiO_2_ sub-micron particle deposition mixture, solution II was 1% water–ethanol solution, and mixtures were incubated for different durations, 10 min, 3 h, 12 h, and 24 h (see Fig. S1†). For modified TiO_2_ monolith deposition mixture, solution II was 5% water–ethanol with 0.005 M of the ligand, and the mixture was incubated for 1 h. The approaches used to characterize the TiO_2_ structures are described in the ESI.†

### Animals and brain sample preparation

Inbred C57BL/6 mice of both genders around the ages of 8 months (young) or 24 months (old) were used. Animals were housed under a 12 h : 12 h light–dark cycle at 18–26 °C, 30–70% humidity. Food and water were accessible *ab libitum*. All animal-related procedures, including euthanasia by cervical dislocation, were performed in compliance with local and federal regulations and according to animal use protocols approved by the University of Illinois Institutional Animal Care and Use Committee.

After quick extraction, brains were chilled at 4 °C in an artificial cerebral spinal fluid tissue slicing solution containing (in mM): 93 *N*-methyl-d-glucamin, 2.5 KCl, 1.2 NAH_2_PO_4_, 30 NaHCO_3_, 20 HEPES, 25 glucose, 5 sodium ascorbate, 2 thiourea, 3 sodium pyruvate, 10 MgSO_4_, and 0.5 CaCl_2_, conditioned with 95% O_2_/5% CO_2_ (adapted from a prior report^50^). Coronal brain slices, 400 μm thick, were cut through the hippocampus and prepared with a Leica VT1000 S vibrating blade microtome (Leica Biosystems, Buffalo Grove, IL). Brain slices were transferred to glass slides covered with Parafilm M (Pechiney Plastic Packaging, Inc., Batavia, IL) and frozen over dry ice for further cryo-sectioning and MS analysis.

### Sample preparation for MSI

The 400 μm thick frozen coronal brain slices were further cut into a set of 10 μm thick sections using a cryostat (Leica CM3050 S, Leica Biosystems Inc.). Sections were thaw-mounted on indium-tin-oxide coated glass slides (Delta Technology Ltd, Loveland, CO) and dried in an N_2_-filled desiccator for 20 min. For TiO_2_-assisted LDI, all types of TiO_2_-containing mixtures were applied onto tissue sections by airbrush-assisted deposition. Briefly, 5 mL of the above described, appropriately diluted TiO_2_-containing solutions were sprayed with a 0.2 mm nozzle airbrush (Paasche Airbrush Company, Chicago, IL), with a nozzle-to-target distance of ∼50 cm and nozzle nitrogen gas pressure set at 35 psi. The final TiO_2_ material layer was ∼400 μg cm^–2^, determined by comparing sample weight before and after sublimation.

For the MALDI MS experiments, DHB was applied onto tissue slices by sublimation, carried out using a laboratory-constructed system similar to one previously described,^51,52^ with some modifications.^51,52^ The sublimation procedure is described in the ESI.† The final matrix layer was ∼500 μg cm^–2^, determined by comparing sample weights before and after sublimation.

### MS analyses

MALDI time-of-flight (TOF)/TOF MSI was performed using an ultrafleXtreme II mass spectrometer (Bruker Daltonics, Billerica, MA) equipped with a solid-state UV Smartbeam II laser. The MSI of tissues was performed using two spatial resolution instrument settings: (1) the “Ultra” laser size setting, with a ∼100 μm diameter footprint and a 100 μm raster step, was used for low spatial resolution MS imaging, and (2) the “Small” laser size setting, with a ∼20 μm diameter laser footprint and a raster step of 30 μm, was used for MSI at high spatial resolution. Due to the large size of the resulting data file and relatively long acquisition time (2 h) for each image, only the hippocampus areas were imaged in the high spatial resolution MSI experiments, whereas a larger brain region was imaged for the lower resolution experiments. Each pixel in the resulting MS images corresponds to a signal in a single mass spectrum acquired as a sum of 1000 laser shots. The laser settings were: 1000 Hz repetition rate, 70% intensity, and a global attenuator offset of 30%. MALDI MS spectra were acquired in the *m*/*z* range of 20–3000. A mixture of DHB, bradykinin, and angiotensin II was deposited near to the brain tissue section for mass spectra recalibration, which was needed to address some cases of surface topography-related molecular mass errors. MS image data acquisition, processing, and visualization were performed with flexImaging software (Version 3.0, Bruker Daltonics). The MALDI TOF/TOF mass analyzer was calibrated with the Peptide Calibration Standard II set (Bruker Daltonics) containing bradykinin, angiotensin II, angiotensin I, substance P, bombesin, ACTH clip 1–17, ACTH clip 18–39, renin substrate, and somatostatin 28.

Tandem MS (MS/MS) analysis was used to elucidate the structural properties of the detected compounds. MS/MS was carried out in the LIFT mode utilizing argon as a collision gas and a 2 Da precursor isolation window.

Equipped with a MALDI ion source, a solariX™ XR Fourier transform ion cyclotron resonance (FTICR) mass spectrometer (Bruker Daltonics) was used for high mass resolution and accuracy measurements of the samples previously analyzed *via* MSI. Measurements were performed with the UV Smartbeam II laser set at 50% laser power and medium size laser footprint. Mass spectra were acquired as a sum of 200 laser shots at 500 Hz laser frequency. The *m*/*z* range of 20–2000 was investigated with an average accumulation number = 4. Mass spectrometer calibration was performed in tuning mode using the Peptide Mixture II Calibration set.

The methodological details of diffuse reflectance UV-vis spectrometry, UV-vis absorption spectroscopy, and electron microscopy measurements, as well as the statistical analysis, are presented in the ESI.†

## Results and discussion

### Detection of lipids with TiO_2_-assisted LDI MS

#### Effect of TiO_2_ particle size on intact lipids and their fragment signals

TiO_2_ nanoparticles were synthesized using the sol–gel method described in a previous study.^33^ Nanoparticle solutions were made by diluting the hydrolyzed sol solutions with ethanol. Almost no intact lipid signals in the *m*/*z* range of 500–1000 were observed when 500 μg cm^–2^ of TiO_2_ nanoparticles were applied on samples (Fig. S1B†). In contrast, intense peaks of characteristic lipid fragments at *m*/*z* 163, 141 and 137 were observed (Fig. S1C†). No lipid signals were observed when different amounts (100 μg cm^–2^, 500 μg cm^–2^, and 2000 μg cm^–2^) of TiO_2_ nanoparticles were applied on brain tissues (Fig. S2†).

Particle size has an effect on the observed lipid signals.^34^ Therefore, to generate TiO_2_ particles of different sizes, the sol solutions were diluted with the ethanol–water reaction solutions (solution II), and the resulting diluted mixtures were incubated for different times to allow further hydrolysis and condensation. With incubation times equal to or below 3 h, larger particles (500–700 nm in diameter) were formed, along with nanoparticles (Fig. S1A-1–A-3†); lipids were detected with the larger TiO_2_ particles but no lipid signals were observed when using the TiO_2_ nanoparticles (Fig. S2†). With a 12 h incubation time, only 500–700 nm TiO_2_ particles were formed, with no nanoparticles detected (Fig. S1A-4†), and resulted in further increase of the intact lipid signals (Fig. S1B†). No further increases in TiO_2_ particle sizes or lipid signals were observed with an incubation time of 24 h. However, signals for the head group fragments of hydrolyzed lipids gradually decreased (Fig. S1B†) with increased TiO_2_ particle sizes, while no similar significant changes in the signal intensities of other small molecules were observed. These results indicate that catalytic hydrolysis of phospholipids on the surface of TiO_2_ particles is responsible for the absence of intact lipid signals during TiO_2_ nanoparticle-assisted LDI MS analysis, which is supported by other studies.^53–55^ Lipid hydrolysis was suppressed with increased TiO_2_ particle sizes. TiO_2_ particles with 500–700 nm diameters are referred to as TiO_2_ sub-micron particles in the following text.

#### Effect of pH on lipids and their fragment signals

Different environmental pH levels will result in different coordination states of Ti sites. With a high pH, the Ti site is coordinated by OH^–^, which may decrease Ti's electrophilicity and its catalytic activity.^43^ In contrast, a low pH will increase the number of Lewis acid sites and facilitate catalysis.

Two inorganic acids, nitric acid (Fig. S3A†) and phosphoric acid (Fig. S3B†), were evaluated at different concentrations as additives to TiO_2_ sub-micron particle-containing solutions (Fig. S3†). A higher concentration of HNO_3_ led to lower lipid signal relative peak areas (RPAs) but higher RPAs of the corresponding lipid head group signals (Fig. S3A†). However, the addition of phosphoric acid, which is a known Lewis base with strong affinity to TiO_2_, led to increased lipid signal RPAs and a decreased ratio of the head group signal RPAs to intact lipids (Fig. S3B†). A Tukey test was performed to determine the statistical significance of the changes (Table S1†). These results can be explained by a decrease in the electrophilicity of the Ti site and a reduction in the catalytic hydrolysis due to coordination of the phosphoric ligands to the Ti site.^43^ It appears that increasing the pH of the TiO_2_-containing solution and protecting Ti sites with specific ligands are effective in reducing the lipid hydrolysis.

### Effect of ligand additions on TiO_2_-assisted LDI MS performance

Avoiding the use of nanoscale TiO_2_ particles can reduce the hydrolysis of lipids; however, the lower surface area of sub-micron particles may decrease the UV absorption of the TiO_2_ material and its adsorption capacity for analytes, which will decrease the detection sensitivity of TiO_2_-assisted LDI MS analysis. To increase sensitivity, the modification of TiO_2_ material with organic ligands was investigated.

#### Selection of organic ligands

**Fig. 1:**
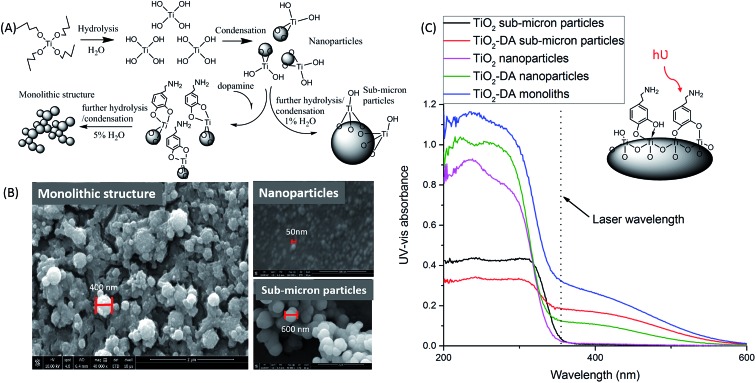
Influence of the addition of DA on the morphology of the TiO_2_ structure and its UV-vis light absorbance. (A) Schematic illustration of the formation mechanism of the TiO_2_-DA monolithic structure.^59^ (B) Scanning electron microscopy images of (left) TiO_2_-DA monolith, (upper right) TiO_2_ nanoparticles, and (bottom right) TiO_2_-DA sub-micron particles. (C) Diffuse reflectance UV-vis spectra of unmodified TiO_2_ particles, and modified TiO_2_ particles and monolith, produced with different methods.

We tested a number of reported bidentate binding ligands for TiO_2_: SA, alizarin, ascorbic acid, and DA.^39^ A large red-shift in the UV spectra of TiO_2_ was observed when the ligands were added into a TiO_2_ nanoparticle solution with a molar ratio of 1 : 10, resulting in an increase in UV absorption of TiO_2_ at a laser wavelength of 355 nm (Fig. S4†). Based on prior studies,^39,56^ ring structures involving five or seven atoms possibly formed during binding (Fig. 1A and C), and the red shift was due to the formation of significant dipole moments in the TiO_2_ particles after the binding.^39^ DA provides the highest dipole moment (16.1 Debye (ref. 39)) and a large shift in UV absorption at 355 nm.

For the MSI evaluation, bidentate binding ligands were added into a TiO_2_ sub-micron particle solution with a molar ratio of 1 : 10, and the resulting solutions were airbrushed onto mouse brain tissue slices. The acquired MS data demonstrate that the intact lipid to lipid fragment peak area ratios for *m*/*z* 844.5: fragment *m*/*z* 141.0, and the intact lipid *m*/*z* 844.5: fragment *m*/*z* 162.9, are much lower with SA-modified TiO_2_ sub-micron particles than with the other additives tested (Fig. S5B†). As discussed in the prior section, SA, a Brønsted acid, likely reduces the pH of the TiO_2_ surface, facilitating the catalysis of lipid hydrolysis by TiO_2_. Additionally, the use of ligands with UV absorption at 355 nm, such as SA and alizarin, for TiO_2_ sub-micron particle modification led to the detection of intense background signals (Fig. S5A†). Strong desorption and ionization of ligands, their fragments, and different complexes can be responsible for the formation of those background signals. In contrast, the addition of DA, which is a Brønsted base, was most efficient in reducing lipid hydrolysis without significant complication of the mass spectra with background peaks. Thus, we selected DA as the bidentate binding ligand for TiO_2_ because it provided the highest UV absorption, a basic environment, and reduced lipid hydrolysis.

To investigate the kinetics of the coordination reaction involved in the formation of TiO_2_-DA bidentate complexes, UV spectra from solutions of TiO_2_-DA nanoparticles with different reaction times were acquired (Fig. S6A†). A time curve of UV absorbance at 355 nm (Fig. S6B†) demonstrates that reaction equilibrium can be reached in 30 min; therefore, a 1 h incubation time for TiO_2_ modification was selected.

**Fig. 2:**
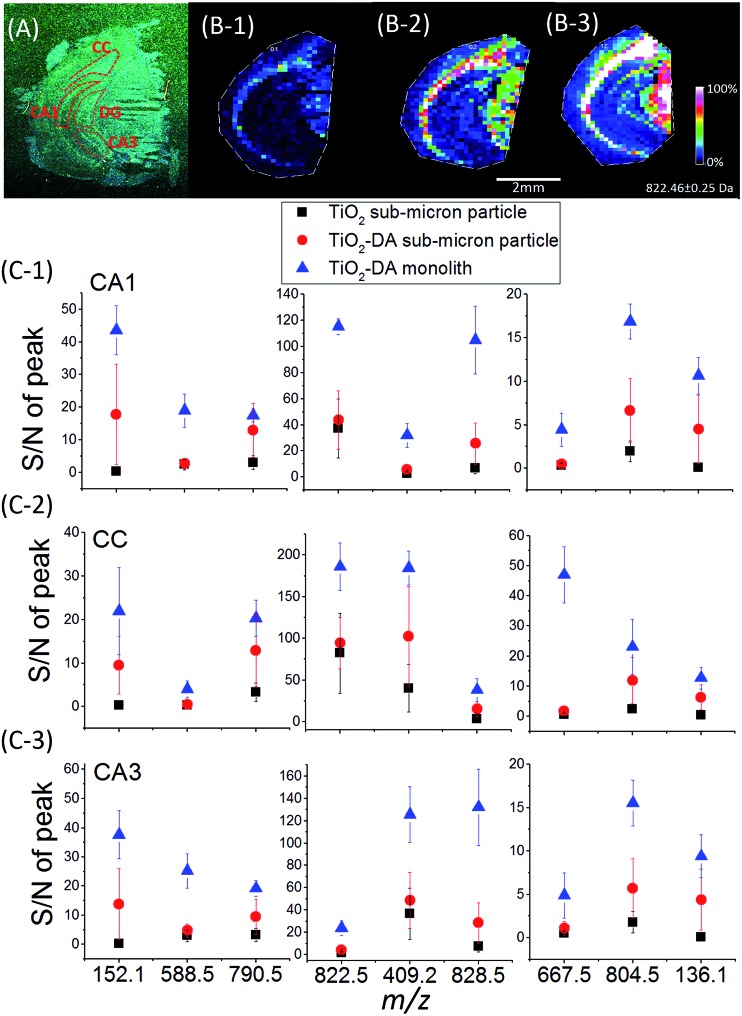
Sensitivity of MSI analyses for endogenous metabolites using different TiO_2_ materials. (A) Optical image of a mouse coronal brain hemisphere section. The anatomical regions are outlined in accordance with existing brain maps.^64^ (B) Ion images of a representative lipid at *m*/*z* 822.5 (PE(p-40:2)) acquired from the mouse brain hemisphere using LDI MS assisted by (1) unmodified TiO_2_ sub-micron particles, (2) TiO_2_-DA sub-micron particles, and (3) TiO_2_-DA monolith. (C) Averaged S/Ns of molecular signals detected in the different brain regions using the three methods mentioned above (*n* = 3; *p* values of the Tukey test are listed in Table S3†). CA1, region I of hippocampus proper; CA3, region III of hippocampus proper; CC, corpus callosum.

A comparison of the mass spectra acquired from mouse brain sections using TiO_2_ and TiO_2_-DA sub-micron particle-assisted LDI MS revealed that similar sets of signals were detected (Table S2†) with both materials, with some exceptions. Additional signals were observed with TiO_2_-DA sub-micron particle-assisted LDI MS, including the Na and K adducts of DA as well as oxidized DA (DA-*o*-quinone),^57^ and also several putatively identified amino acids and dipeptides obtained by matching measured and theoretical accurate molecular masses (representative mass spectra are shown in Fig. S7†). Dipeptides may form in the mass spectrometer source by photocatalytic redox reactions, resulting in fragmentation of longer peptides.^58^ Importantly, except for the additional signals mentioned above, the LDI MS using TiO_2_-DA sub-micron particles resulted in up to a 10-fold increase in the signal-to-noise ratios (S/Ns) for peaks at *m*/*z* 200–1000 in mouse brain tissue compared to untreated TiO_2_ sub-micron particles, without changing their spatial distribution (Fig. 2C).

#### Preparation and application of TiO_2_-DA monolith

Despite the successful use of TiO_2_-DA sub-micron particles in LDI MS, their relatively low surface area, which is also affected by the particles self-clustering (Fig. 1B), reduces the number of DA-bound Ti^4+^ sites. To increase the number of bound Ti^4+^ sites, DA was added into the reaction solution (solution II) before the TiO_2_ sol solution (solution I) was added, and prior to the formation of sub-micron particles. The high surface area of the TiO_2_ nanoparticles led to a higher number of active Ti^4+^ sites that were available for DA binding. However, the bidentate binding of Ti^4+^ with DA decreased the rate of the condensation reaction. Since slow hydrolysis and fast condensation facilitate the formation of TiO_2_ particles, the attenuated condensation rate suppressed the growth of TiO_2_ particles^59–61^ (Fig. 1A). LDI MSI was used to assess the effect of various percentages of water content in the reaction solution with DA added. With 1% water, mostly nanoscale particles were generated (Fig. S8A†), resulting in relatively low lipid signals acquired from mouse brain sections. When the water content was increased to 2.5% (Fig. S8B†), particle sizes also increased, and the resulting sub-micron particles had a rougher morphology and tended to aggregate together with better uniformity than the TiO_2_ sub-micron particles formed without DA; this led to an increase in the lipid signals. Raising the water content to 5% led to a greater aggregation of TiO_2_-DA sub-micron particles, generating loose skeletons, and even stronger lipid signals were detected using these structures (Fig. S8C†). The TiO_2_-DA macroporous skeleton structures are similar to the monolithic structures that are widely used in the field of materials science.^59,62,63^ A high concentration of chelating ligands and optimized water content in the reaction solution are key factors in the successful formation of monolithic structures.^59–61^ As shown in Fig. 1A, bidentate binding by DA attenuated the condensation of the titanium precursor, but higher water content led to fast hydrolysis and potentiated the increase in the number of hydrolyzed Ti–OH cores, facilitating the formation of a three-dimensional network of TiO_2_. The lipid signals did not further increase when the water content was raised to 10% (Fig. S8D†) Thus, 5% water proved optimal for the following experiments. Moving forward in our discussion, TiO_2_ material with this special structure is referred to as the TiO_2_-DA monolith.

We assessed the sensitivity of our LDI MS analyses using the various TiO_2_ materials (Fig. 2). With the TiO_2_-DA monolith, the S/Ns of most of the peaks in the range of 100–1000 *m*/*z* increased between 10- to 30-fold as compared to the results with unmodified TiO_2_ sub-micron particles. In addition, a lot of compounds with average S/Ns below the detection limit when using unmodified TiO_2_ were detected with S/Ns up to 40 with the TiO_2_-DA monolith. These improvements in the S/Ns are attributed to the higher surface area and higher concentration of bound Ti sites within the TiO_2_-DA monolith. The diffuse reflectance UV-vis spectroscopy analysis of TiO_2_ and TiO_2_-DA materials shown in Fig. 1C demonstrates that such changes lead to an increase in the UV absorption efficiency of the materials. The diffuse reflectance UV-vis spectrum of a TiO_2_-DA sub-micron particle has a red shift compared with that of an unmodified TiO_2_ sub-micron particle. The TiO_2_-DA monolith has a much higher UV absorbance compared to the DA-modified TiO_2_ sub-micron particles.

In addition to improving the analyte detection sensitivity, the hydrolysis of lipids on TiO_2_-DA materials was also investigated. The ratio of peak areas for a representative lipid signal and one characteristic fragment signal was calculated (Fig. S9†). The ratios are higher with the TiO_2_-DA-modified materials than the unmodified materials. However, the ratios are similar when comparing datasets obtained with TiO_2_-DA sub-micron particle- and TiO_2_-DA monolith-assisted LDI MS. The data indicate that the presence of DA in the TiO_2_ sub-micron particles and monolith can lead to lower lipid hydrolysis. This can be explained by the fact that DA binding can decrease the electrophilicity of the Ti site.

Moreover, it can be seen from the scanning electron microscopy images (Fig. 1B and S10†) that the TiO_2_-DA monolith layer is more uniform and provides high sample surface coverage (93%); the coverage area was calculated using Adobe Photoshop CC (2014). This uniformity can be also be beneficial in high spatial resolution MSI analysis. After deposition on the TiO_2_ gel sample surface, small visible cracks developed in the coating (Fig. S10†), likely caused by shrinkage during drying. Since the crack widths (<1 μm) are much smaller than the imaging raster gap (30–100 μm) and the laser's footprint size, these cracks should not affect the spatial resolution of the MSI, as discussed further in next section.

#### Repeatability of TiO_2_-DA monolith-assisted LDI MS

Hydrolysis reactions are sensitive to temperature and humidity, which are difficult to control precisely during typical biological sample preparations for MSI measurements. To test the repeatability of TiO_2_ sub-micron particle-, TiO_2_-DA sub-micron particle-, and TiO_2_-DA monolith-assisted LDI MS measurements, we conducted a series of MSI experiments. Samples collected from three different mice were analyzed separately in three different weeks. Three adjacent slices from each animal's brain were measured as intraday technical replicates. The general trend of the increase in the S/N values is shown in Fig. S11† for the data acquired using unmodified TiO_2_ sub-micron particles, TiO_2_-DA sub-micron particles, and TiO_2_-DA monolith. Overall, the S/N profiles for different weeks and different brain regions are repeatable, with several exceptions. The relative standard deviations of 80% of the signals detected using the optimized TiO_2_-DA monolith-assisted LDI MSI method are below 30%, demonstrating acceptable intraday repeatability of the approach. However, a comparison of the S/N of peaks between different weeks revealed more variability in the acquired data, which can be explained by the biological variability of the studied animals, slight differences in sample preparation, and temporal shifts in instrumental performance.

### Comparison of TiO_2_-DA monolith-assisted LDI MSI with traditional MALDI MSI

#### Background signals in TiO_2_-DA-assisted LDI MS analysis

**Fig. 3:**
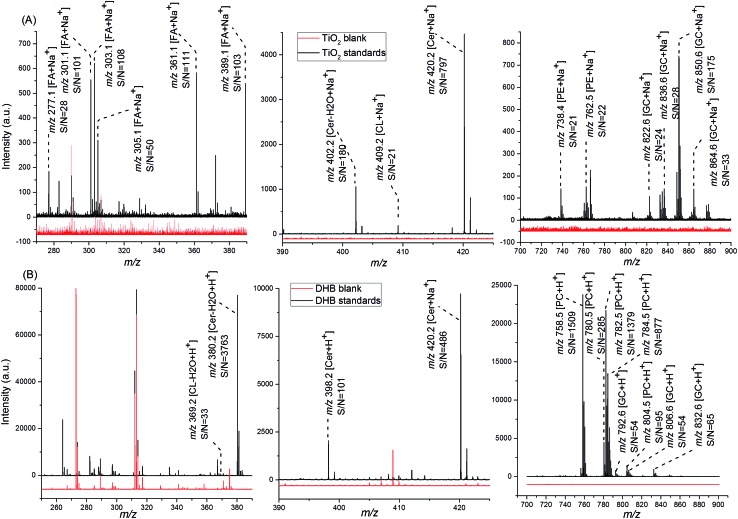
Representative mass spectra of lipid standards detected with (A) TiO_2_-DA monolith-assisted LDI MS and (B) MALDI MS using DHB as the MALDI matrix. Mass spectra in red were acquired from blank samples and mass spectra in black were acquired from samples of lipid standards. FA, fatty acids; Cer, ceramide; CL, cholesterol; PE, phosphatidylethanolamine; PC, phosphatidylcholine; GC, galactocerebroside.

TiO_2_-assisted LDI MS is reported to provide low background noise;^31,33,34^ however, background signals of Ti_*x*_O_*y*_ have been detected when the TiO_2_ nanoparticles are not fully hydrolyzed.^65^ Here, mass spectra obtained from TiO_2_ sub-micron particles, TiO_2_-DA sub-micron particles, and TiO_2_-DA monolith all had predominantly low intensity background peaks (Table S2†). TiO_2_ sub-micron particle-coated blank samples exhibited only three peaks (Table S2†). A few small background signals in the low *m*/*z* range were found in the mass spectra of TiO_2_-DA monolith blanks (Table S2†) however, these signals were mostly suppressed when TiO_2_-DA-based materials were used in our biological sample analysis. In contrast, mass spectra from the DHB blank samples contained a large number of MALDI matrix-related peaks with *m*/*z* below 500 Da (Fig. 3B). Therefore, the TiO_2_-DA monolith provides a higher peak capacity for the detection of small analytes in complex samples.

#### Selectivity and sensitivity of TiO_2_-DA monolith-assisted LDI MS

TiO_2_ has selective affinity to Lewis bases, such as amino groups, enediol groups, carboxyl acidic groups, and phosphate groups.^38–40^ For example, TiO_2_-assisted LDI MS allows detection of r-cyclodextrin and catechins, both containing Lewis basic groups.^29,32^ In order to compare the selectivity and sensitivity of TiO_2_-DA monolith-assisted LDI MS and traditional MALDI MS using DHB, we used lipid standards with different functional groups. The standards mixture consisted of the carboxyl group containing fatty acids, the 1,3-propanediol group containing *N*-hexanoyl-d-sphingosine, the 1,2-enediol group containing galactocerebrosides, and the hydroxyl group containing cholesterol as well as PCs and phosphatidylethanolamines (PEs) exhibiting amino groups and quaternary amine. As shown in Fig. 3A, PE but not PC signals were detected with TiO_2_-DA monolith-assisted LDI. In contrast, intense PC peaks dominate the mass spectra acquired by MALDI MS (Fig. 3B). These results demonstrate that the TiO_2_-DA monolith has a selective affinity for Lewis basic groups, making detection of many PCs difficult. In contrast, DHB-based MALDI MS in positive detection mode did not exhibit such selectivity for PEs, and so PCs with the highly ionizable choline cationic head group were detected. MALDI MS measured *N*-hexanoyl-d-sphingosine, cholesterol, and galactocerebroside as [M + H]^+^ or [M – H_2_O + H]^+^ ions, while TiO_2_-DA monolith-assisted LDI MS predominantly detected [M + Na]^+^ and [M + K]^+^ ions. The corresponding ion signals have similar S/Ns (Fig. 3); however, for the DHB-based TOF MS measurements, some of the galactocerebroside peaks overlap the PC peaks with the mass resolution of our measurements. Since PCs are a major component of biological membranes and have high abundance in brain samples, detection of low-abundance lipids is difficult when using the traditional MALDI matrix DHB. In contrast, TiO_2_-DA monolith-assisted LDI MS is more selective and sensitive to low-abundance lipids with Lewis basic groups. Finally, only TiO_2_-DA monolith-assisted LDI MS detected fatty acids. Thus, the use of the TiO_2_-DA monolith in LDI MS measurements may broaden the coverage of lipids and facilitate low-abundance lipid detection, which are important capabilities in lipidomics and cell signaling research.

### TiO_2_-DA monolith-assisted LDI MSI

#### TiO_2_-DA monolith-assisted LDI MSI for investigation of the mouse brain

The mouse brain, one of the best studied neurobiological models, is well-suited for MSI method development and validation. A substantial body of information on the structural, functional, and biochemical parameters of the mouse brain has been collected, especially for the hippocampus, which plays an important role in the mechanisms of memory. Here, we utilized TiO_2_-DA monolith-assisted LDI MSI to investigate the spatial variation in the chemical composition of the mouse hippocampus.

**Fig. 4:**
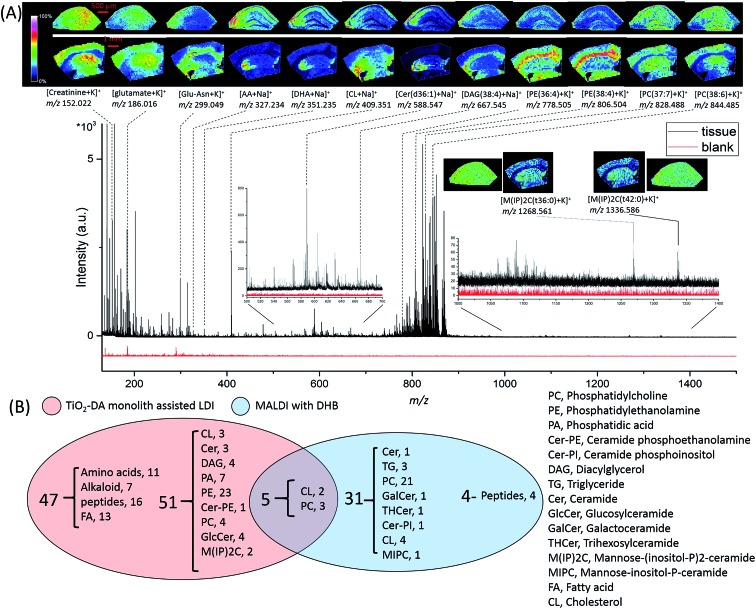
Performance of TiO_2_-DA monolith-assisted LDI MSI of the mouse brain (A) representative MS spectra and associated ion images of selected analyte distributions in the hippocampal region of the mouse brain acquired using TiO_2_-DA monolith-assisted LDI MSI. The upper and lower rows of the ion images were acquired at high (30 μm raster step size) and low (100 μm raster step size) spatial resolutions, respectively. The high resolution images were generated from the hippocampus only. The low spatial resolution images were acquired from slightly larger brain regions, which included the hippocampal areas. (B) Venn diagram illustrating the classes and numbers of identified or putatively identified molecules detected with TiO_2_-DA monolith-assisted LDI MSI and MALDI MSI with DHB as the MALDI matrix, both operating in positive mode.

A large number of signals were detected in the mass range of 100 to 1500 Da in hippocampal samples using TiO_2_-DA monolith-assisted LDI MSI (Fig. 4). Detected compounds were identified with different levels of confidence by comparing metabolite information available in the online databases (METLIN, HMDB, and LIPID MAPS) with one or a combination of several measured analyte characteristics, including accurate molecular mass determined with high mass resolution FTICR MS and molecule fragmentation patterns acquired using MALDI-TOF/TOF MS/MS (Fig. S12 and Table S4†). For example, the identity of the detected PE lipids was determined by their presence in the MS/MS spectra of these lipid fragment signals, with *m*/*z* 180 and *m*/*z* 141 corresponding to the potassiated and monoprotonated HPO_3_C_2_H_4_–NH_2_ head group, and characteristic of the C_2_H_4_–NH_2_ neutral loss of 43 Da (Fig. S12A†). PCs were identified by observation of a neutral loss of 183 Da, corresponding to the detachment of the HPO_3_C_2_H_4_–N(CH_3_)_3_^+^ head group, and the neutral loss of 59 Da, matching the *m*/*z* N(CH_3_)_3_ fragment ion (Fig. S12B†). The sphingoid base ion at *m*/*z* 265 is characteristic for ceramides (Fig. S12E†). The 384 Da loss corresponds to the detachment of the mannose–phosphate–inositol group, characteristic for fragmentation of mannose–(inositol-P)2-ceramide (M(IP)2C) molecules (Fig. S12C†). Assignments of the fragment identities for other molecules are shown in (Fig. S12D and F–J†). When the fragmentation of targeted analytes was not conclusive, assignments of molecular identities were done by matching detected and predicted *m*/*z* values with the lowest mass error (Δppm). For candidates with the same Δppm, compounds that have been previously reported in animal tissues and organs, including the brain, were chosen and listed in Table S4.† The presented identifications of the detected molecules have different levels of confidence.

Molecules detected in the *m*/*z* range of 100 to 500 Da are categorized as amino acids, peptides, alkaloids, fatty acids, and cholesterols (Table S4†). In the molecular mass range of 500 to 700 Da, most of the detected molecules are ceramides and diacylglycerols (DAGs). The mass range of 700 to 1200 Da is populated by phospholipids, including PEs and PCs. The use of TiO_2_-DA monolith-assisted LDI MSI allowed detection of both PEs and PCs, with PEs dominating the mass spectra. Several low intensity M(IP)2C signals were observed in the mass range of 1000 to 1500 Da. In contrast, positive mode MALDI MS detected predominantly PC species. Analysis of the data acquired with TiO_2_-DA monolith-assisted LDI MS revealed that 103 molecules were detected and identified with different levels of confidence using this approach; in contrast, only 40 endogenous molecules could be characterized with DHB-based MALDI MSI (Fig. 4B). Even with negative mode MALDI MS using the MALDI matrix 9-AA, only 6 PEs, 3 phosphatidylserines and 9 sulfatides were identified (Table S4†). A literature search suggests that 19 of the 103 compounds observed have not been detected previously with MALDI MS or LDI MS, but have been measured with other MS methods in mouse and rat brains (corresponding literature citations are labeled “c” in Table S4†). Moreover, to the best of our knowledge, 30 of the compounds detected here have not been reported in the mouse brain with other MS methods (citations are labeled “d” in Table S4†).

Acquired ion images with low (100 μm) and high (30 μm) spatial resolutions depict localizations of different compounds, including lipids, in specific sub-regions and cell layers of the hippocampus (Fig. 4A).

#### Investigation of chemical manifestations of brain aging using TiO_2_-DA monolith-assisted LDI MS

**Fig. 5:**
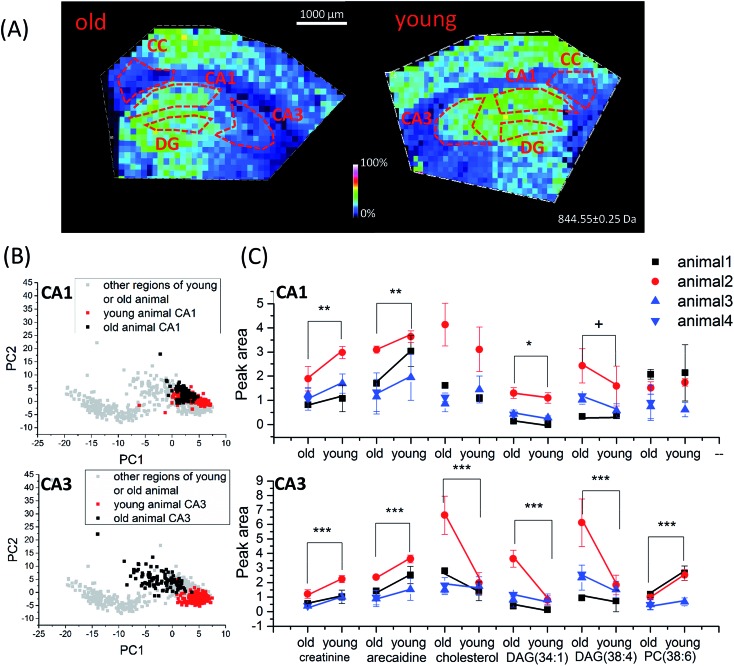
Age-dependent spatio-chemical differences in the hippocampus detected with TiO_2_-DA monolith-assisted LDI MSI. (A) Representative ion images of cholesterol (*m*/*z* 409.3). (B) PCA score plots of TiO_2_-DA monolith-assisted LDI MSI datasets acquired from different hippocampal and surrounding regions of young and old mice. (C) Statistical comparison of average peak areas of identified molecular signals (data points acquired from the same batch of old and young animal samples are marked by the same color). *p* values for datasets collected from animals of different ages; samples were calculated using two-way ANOVA, and related detailed information is provided in Table S5.† ***, *p* value < 0.001, **, *p* value < 0.01, *, *p* value < 0.05, +, *p* value < 0.1. CA1, region I of hippocampus proper; CA3, region III of hippocampus proper; DG, dentate gyrus; CC, corpus callosum.

Chemical changes associated with brain aging have been extensively studied.^43,45,66–70^ Aging affects brain functions such as learning and memory,^48^ and coincides with a number of debilitating disease conditions, including Alzheimer's disease.^44,71,72^ Information processing by the hippocampus is critical to memory formation and recall.^73^ The layered morphological organization of the hippocampus is well characterized, providing robust structural references for chemical imaging. Using TiO_2_-DA monolith-assisted LDI MSI we can explore age-dependent spatio-chemical changes in the mouse brain in a multiplexed manner. In this work, a comparative analysis of samples collected from three 8 month old mice and four 2 year old mice was performed. Two to four technical replicates from each of the collected brain tissues were performed. The main focus of the measurements was to compare the chemical analyses of different hippocampal subregions, including the CA1, CA3, dentate gyrus, and the corpus callosum, in animals of different ages (Fig. 5A). The multiplexed datasets acquired from the selected hippocampal regions were evaluated with PCA, a complementary multivariate analysis technique. PCA decreases the dimensionality of the datasets and finds main differences between them, with the data presented as score plots (Fig. 5B and S13†). Age-dependent chemical differences of the CA1 and CA3 regions determined by principal component 1 (PC1) are shown in Fig. 5B. The loading plot for corresponding datasets revealed which compounds contributed most to the observed differences (Fig. S14†). Our data correlate with previously published information for a number of molecules. For example, compounds with high PC1 values in the loading plots correspond to known animal aging biomarkers, such as GABA (*m*/*z* 142),^45^ glutamate (*m*/*z* 186),^45^ creatinine (*m*/*z* 152),^66^ cholesterol (*m*/*z* 409.3),^43^ DAGs (*m*/*z* 500–600 range),^67–70^ and PCs/PEs (*m*/*z* 600–900 range).^68,74^ Six molecules were chosen for targeted statistical analysis: creatinine, arecaidine, cholesterol, DAG(38:4), DAG(34:1), and PC(38:6). The average peak areas of signals acquired from the studied brain regions were calculated and evaluated with two-way ANOVA to determine age-dependent differences. The evaluated compound signal peak areas exhibited significant differences between old and young animals in at least one brain region (CA3) analyzed (Fig. 5C). Detailed ANOVA results are provided in Table S5.† ANOVA can distinguish the effects of aging and experimental batch effects of measurements. Interestingly, nearly all of the analyzed lipid signals showed significant increases in aging animal specimens, except PC(38:6). In contrast, the signals of two small metabolites (creatinine and aracaidine) decreased with aging (Fig. 5). The results, such as increased cholesterol and DAG signals, match prior reports.^69,70^ The observed decrease in PC(38:6) signal also agrees with previously published findings.^74^

## Conclusions

TiO_2_-assisted LDI MSI approaches using TiO_2_ materials with various morphologies and surface properties were systematically evaluated. It was found that lipid hydrolysis on a TiO_2_ surface can be largely reduced by increasing TiO_2_ particle size from the nanoscale (<50 nm) to sub-micron scale (>500 nm), and also by increasing the pH of the evaluated TiO_2_-containing suspensions. The sensitivity of TiO_2_-assisted LDI MSI detection for a variety of endogenous molecules was largely increased by modifying TiO_2_ materials with DA, and optimizing the TiO_2_ morphology to be monolithic with DA binding. In comparison with unmodified TiO_2_ sub-micron particle-assisted LDI MS, the peak S/Ns for both small molecule and large lipid signals in the molecular mass range of 100 to 1500 Da were increased up to 30-fold with TiO_2_-DA monolith-assisted LDI MS. By comparing the new method described here with traditional MALDI MS, TiO_2_-DA monolith-assisted LDI MS performed in positive mode demonstrated higher selectivity and sensitivity for Lewis basic lipids, such as fatty acids, cholesterols, ceramides, DAGs, and PEs. Importantly, the approach is less sensitive for the detection of high-abundance PCs. Investigation of age-related chemical changes in the hippocampus of mice confirmed the utility of TiO_2_-DA monolith-assisted LDI MSI measurements for fundamental neuroscience. Thirty-five small molecules (including amino acids, alkaloids, free fatty acids, and dipeptides) and over 50 lipids (including cholesterols, ceramides, DAGs, glucosylceramide, PA, ceramide phosphoethanolamine, PCs, and PEs) were detected with TiO_2_-DA monolith-assisted LDI MSI and identified with MALDI FTICR MS and/or MALDI MS/MS with different levels of confidence. PCA of datasets acquired from brain samples using TiO_2_-DA monolith-assisted LDI MSI identified several different molecules in young and aging mice.

In summary, traditional MALDI matrixes work efficiently in the MSI of mostly large and abundant molecules such as lipids and peptides. Previously reported nanomaterial-assisted LDI, which provides spectra with low background noise, is useful for the detection of small molecules, but may have limitations when characterizing larger compounds and larger sets of chemical classes. TiO_2_-DA monolith-assisted LDI MSI enhances the efficacy of nanomaterials for lipid detection and their high selectivity for Lewis basic compounds. In all, this approach presents an opportunity for the detection of both small (<500 Da) and large molecules (500 to 1000 Da), and complements traditional MALDI MS approaches by providing additional information on the chemical composition of complex biological tissues.
