# Supplementary material for: Dopamine-modified TiO2 monolith-assisted LDI MS imaging for simultaneous localization of small metabolites and lipids in mouse brain tissue with enhanced detection selectivity and sensitivity
Source: Chem Sci. 2017 Mar 21;8(5):3926–38. doi: 10.1039/c7sc00937b (PMC5433501; doi:10.1039/c7sc00937b)
Supplement: Supplementary file 1 [file SC-008-C7SC00937B-s001.pdf]

Electronic Supplementary Material (ESI) for Chemical Science.
This journal is © The Royal Society of Chemistry 2017

Dopamine-Modified TiO2 Monolith-Assisted LDI MS Imaging for Simultaneous Localization of Small Metabolites and Lipids in Mouse Brain Tissue with Enhanced Detection Selectivity and Sensitivity

Qian Wu[1,3], James L. Chu[2], Stanislav S. Rubakhin[1,3], Martha U. Gillette[2,3], Jonathan V. Sweedler[1,3]

[1] Department of Chemistry and the Beckman Institute, University of Illinois at Urbana-Champaign, Urbana, Illinois 61801, United States

[2] Department of Cell and Developmental Biology, University of Illinois at Urbana-Champaign, Urbana, Illinois 61801, United States

[3] Beckman Institute, University of Illinois at Urbana-Champaign, Urbana, Illinois 61801, United States

Supplementary Information

Contents

Additional experimental details S-2
Figures S1–S12 .... S-4
Tables S1–S5. .... S-18
References. S-31

Additional experimental details

Tissue preparation and sectioning

In all cases, the rodent brains were surgically dissected, frozen in liquid nitrogen, and stored at –80 ºC until use. Coronal tissue sections, 14-µm thick, were prepared from frozen mouse cerebrum using a cryostat (3050S, Leica Biosystems Inc., Buffalo Grove, IL) at –19 ºC and thaw-mounted onto conductive indium-tin oxide (ITO)-coated glass slides (Delta Technologies, Loveland, CO).

While most samples were analyzed immediately following preparation, some sections were stored at –80 ºC for later use. Optical images of the tissues were taken using a flatbed scanner (Epson Perfection V300, Epson America, Inc., Long Beach, CA) with a resolution of 2400 dpi before MSI. The optical images shown in Figure 3 were adjusted to aid in visualization of hippocampal structures using Adobe Photoshop 2014.

Materials characterization

UV-vis absorption spectra detection was performed on an EPOCH TM microplate spectrophotometer (Biotek Instruments, Inc., Winooski, VT) with a scanning range of 200–700 nm. TiO_2 nanoparticle sol solution at the concentration of 0.05 M was measured directly by adding 200 µL solutions into a standard 96-well plate.

Diffuse reflectance UV-vis spectrometry was performed on a Cary 5000 UV-Vis spectrophotometer (Agilent, Santa Clara, CA) with a wavelength scanning range of 200–700 nm. The same amounts of different TiO_2 materials were first coated on quartz slides (1" × 1", Ted Pella, Inc., Redding, CA) with an airbrush (Paasche Airbrush Company, Chicago, IL), and then the slides were used for detection, with bare quartz slides evaluated as blanks.

An environmental scanning electron microscope (ESEM) (Philips XL30 ESEM-FEG, FEI, Hillsboro, OR) was used to investigate the micro-morphology of the TiO_2 materials coated on tissue slices. Before ESEM detection, the samples were coated with gold for 1 min with a sputter coater (Desk-1 TSC, Denton Vacuum, Moorestown, NJ).

Laboratory-constructed system for MALDI matrix sublimation

Sublimation was carried out using a laboratory-constructed system, similar to one previously described, with some modifications.[1,2] Briefly, an aluminum foil boat was affixed with double-sided conductive copper tape to the inner base of a sublimation chamber. Samples were attached to a copper plate affixed to the bottom face of an ice-filled cold finger. The MALDI matrix-to-sample distance was ~20 mm. For each sublimation deposition, 350 mg of powdered DHB was distributed evenly in the boat. The sublimation chamber and cold finger were assembled together per manufacturer's instructions, pumped to intermediate vacuum (~10 mTorr), and placed in a heating mantle (Glas-Col LLC, Terre Haute, IN) to equilibrate the vacuum and cool the sample plate. The optimized deposition conditions for derivatized samples included supplying 120 V to the heating mantle for 12 min. After matrix deposition, the chamber was removed from the mantle, vented with room temperature air (25 ºC), and the sample promptly removed from the cold finger.

Data analysis

The molecular ion distribution images of the tissue sections were visualized using flexImaging software 4.1 (Bruker Daltonics, Billerica, MA). MALDI MSI data acquired from triplicate or duplicate brain slices from the same mouse, thaw-mounted on three or two separate slides, were used in the statistical analysis.

For method development, samples from the same mouse were used with different sample preparation conditions on different slides.

In the method application experiments, optimized sample preparation conditions were used in the analysis of specimen from 8-month-old (young, n = 3) and 24-month-old (old, n = 4) mice. Slices from different animals were thaw-mounted on the same ITO slide. Statistical comparisons of peak intensities, areas, or S/Ns acquired from different brain regions of different animals, or samples from the same animal but prepared with different sample preparation conditions, were performed by exporting data from manually-defined regions of interest (ROIs). Mass spectra from each ROI were imported into ClinProTools software 3.0 (Bruker Daltonics) with automatic baseline subtraction and total ion count normalization, except for the data presented in Figure 3 and Figure S5. Peaks were picked with an S/N threshold greater than 3 on average spectra. Picked peak parameters were exported as *m/z* value-peak area tables. To analyze changes in the levels of molecular signals in different subregions of mouse brain, ROIs were chosen. ROIs were outlined using ion maps and optic images aligned to the appropriate Mouse Brain Atlas schematics (http://mouse.brain-map.org/static/atlas). Four ROIs were selected: the CA1, CA3, and dentate gyrus of the hippocampus region, and corpus callosum (shown in Figure 6A). The mass spectra acquired at chosen regions were imported into ClinProTools, set to pick the peaks with an S/N >3. Peak areas were calculated and exported creating a single data set. For the multivariate analysis (principal component analysis, (PCA)) presented in Figure 6, the dataset was imported into OriginPro 8.5 (OriginLab Corporation, Northampton, MA). The mean values of peak areas for signals acquired in selected ROIs were calculated, and the values of technical triplicates or duplicates were used to calculate the relative standard deviation of the replicate measurements. For comparison of the averaged peak areas acquired from different animal groups (old vs. young) described in Figure 5, two way analysis of variance (ANOVA) was performed with OriginPro 8.5 to determine the significance of age effect on compound intensity.

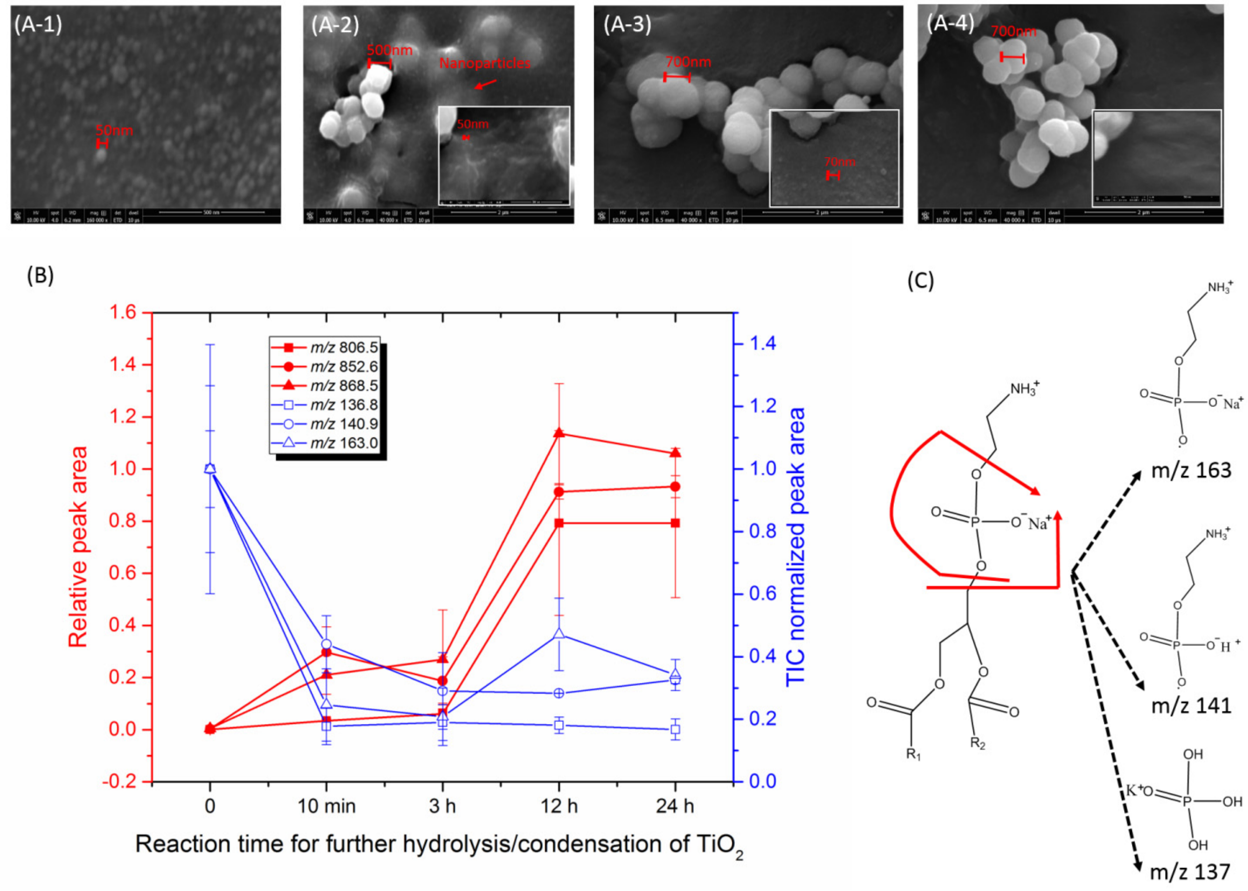

Figure S1. Dependence of TiO_2 particle morphology on (A) reaction time for further hydrolysis/condensation (A-1, 0 min; A-2, 10 min; A-3, 3 h; A-4, 12 h). (B) Relation between TiO_2 condensation time and relative peak area of intact lipid signals (red lines) and lipid fragment signals (blue lines) acquired from brain tissue. (C) Fragmentation of PE and structures of its fragments. n = 2; the p values of a Tukey test for all conditions are presented in Table S5; *m/z* 806.5 and *m/z* 852.6 were identified as $[PE (38:4)+K]^+$ and $[PE(42:9)+K]^+$, respectively, according to FTICR and TOF/TOF MS/MS spectra.

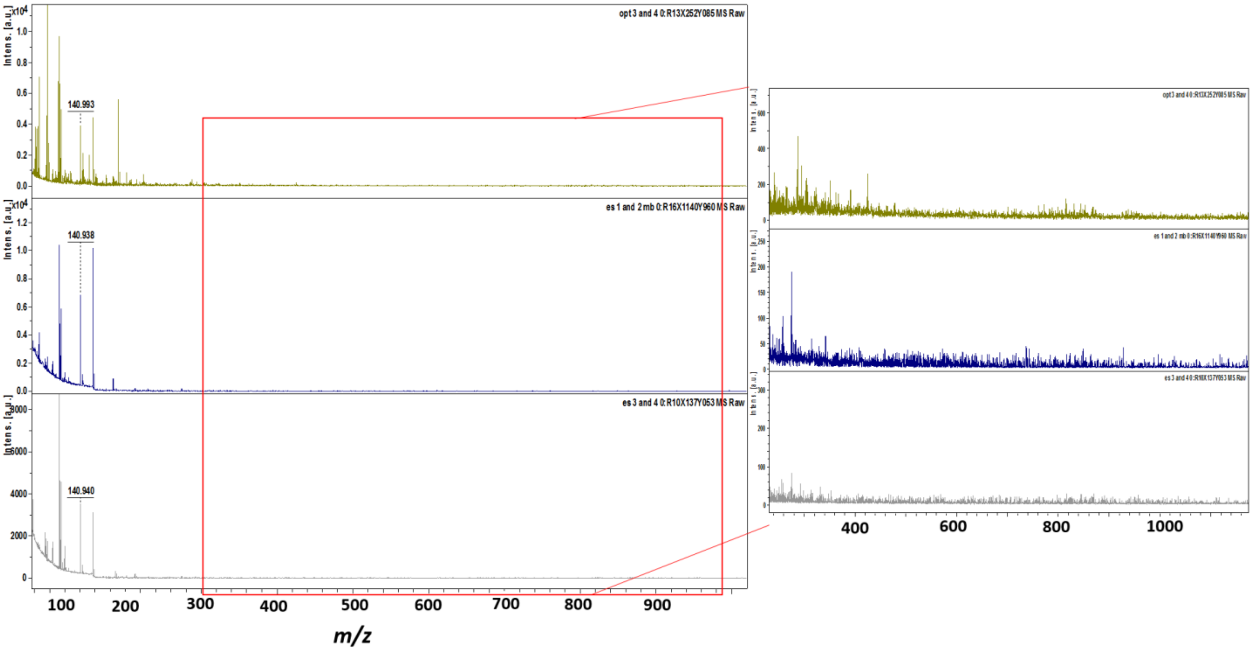

Figure S2. Representative mass spectra acquired from mouse brain tissues using different amounts of TiO_2 nanoparticles. No lipid signals were observed when using the TiO2 nanoparticles. (top) 100 µg/cm^2; (middle) 500 µg/cm^2; (bottom) 2000 µg/cm^2. The inset shows the enlarged spectra for *m/z* 300–800.

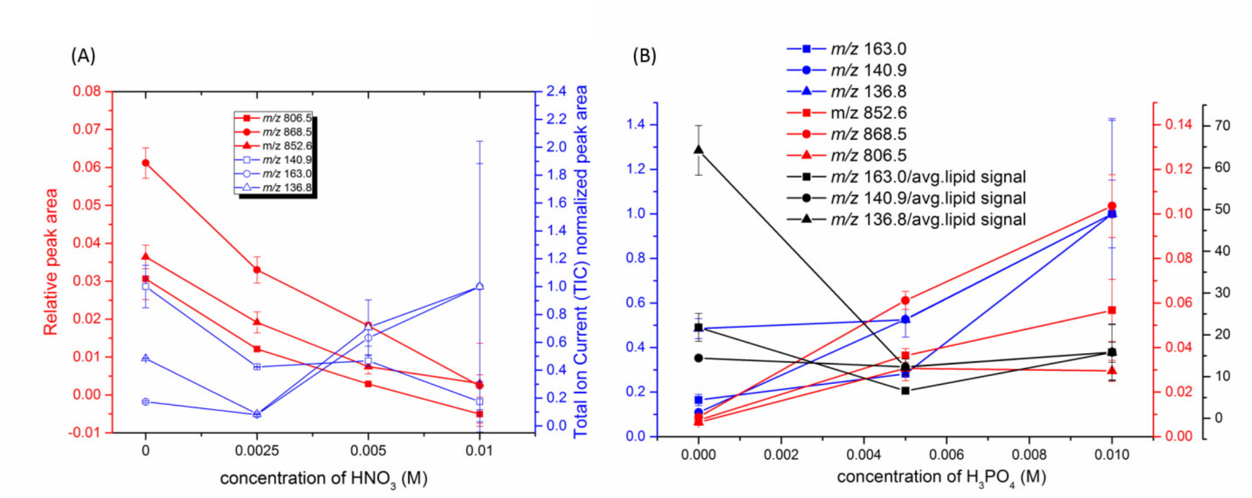

Figure S3. Effects of (A) nitric acid and (B) phosphate acid concentrations in TiO2-sub-micron particle-containing deposition solutions on intact lipid (red lines) and their fragment (blue and black lines) signals acquired with TiO_2-assisted LDI (n = 2). The p values of a Tukey's test for all conditions are listed in Table S3; *m/z* 806.5 and *m/z* 852.6 were identified as $[PE(38:4)+K]^+$ and $[PE(42:9)+K]^+$, respectively, according to FTICR and TOF/TOF MS/MS spectra.

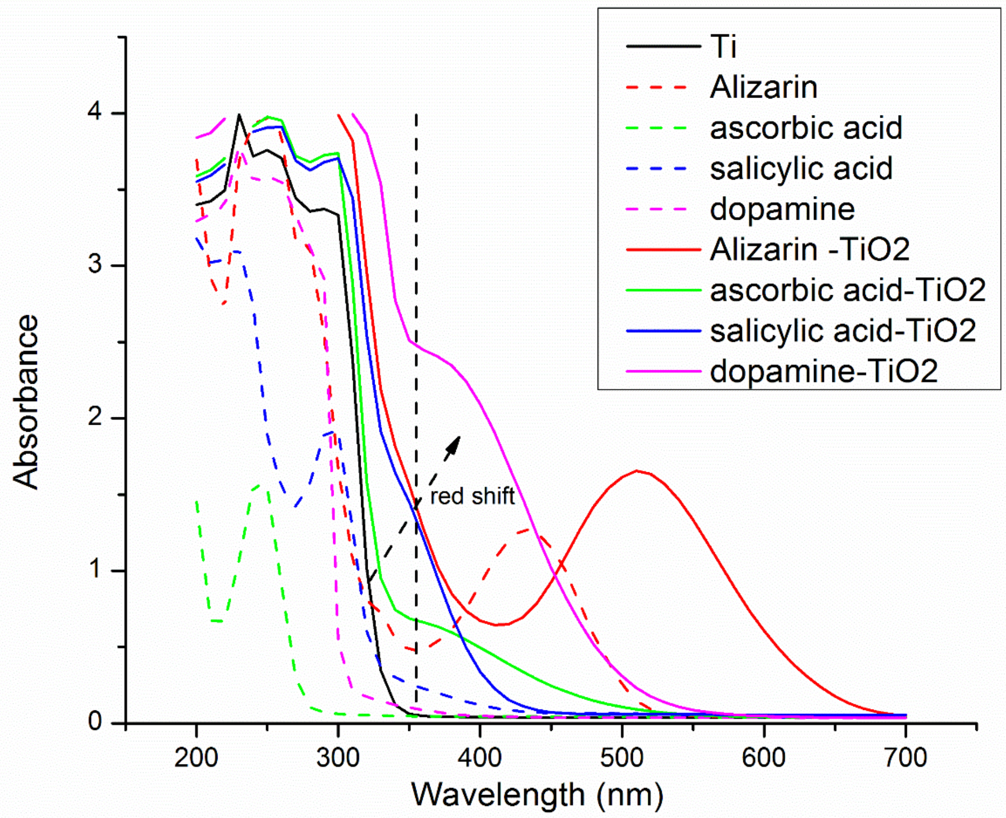

Figure S4. UV-vis spectra of solutions of different bidentate binding ligands, TiO_2 nanoparticles, and bidentate binding ligand-modified TiO_2 nanoparticles.

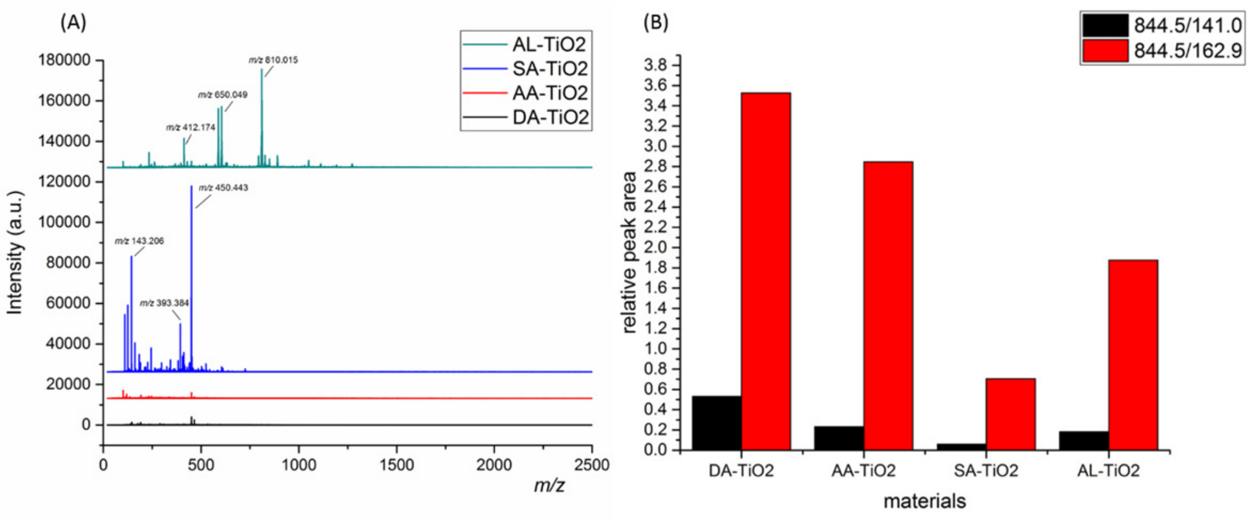

Figure S5. Performance of different chemical modifications of TiO_2 sub-micron particles in LDI MS measurements of region I of the hippocampus proper area of the mouse brain. (A) Mass spectra acquired from modified TiO2 sub-micron particle blank samples. (B) Relative peak area ratios of the intact lipid (*m/z* 844.5) compared to its fragments (*m/z* 141.0 and *m/z* 162.9) using the different modified particles. AL, alizarin; SA, salicylic acid; AA, ascorbic acid; DA, dopamine.

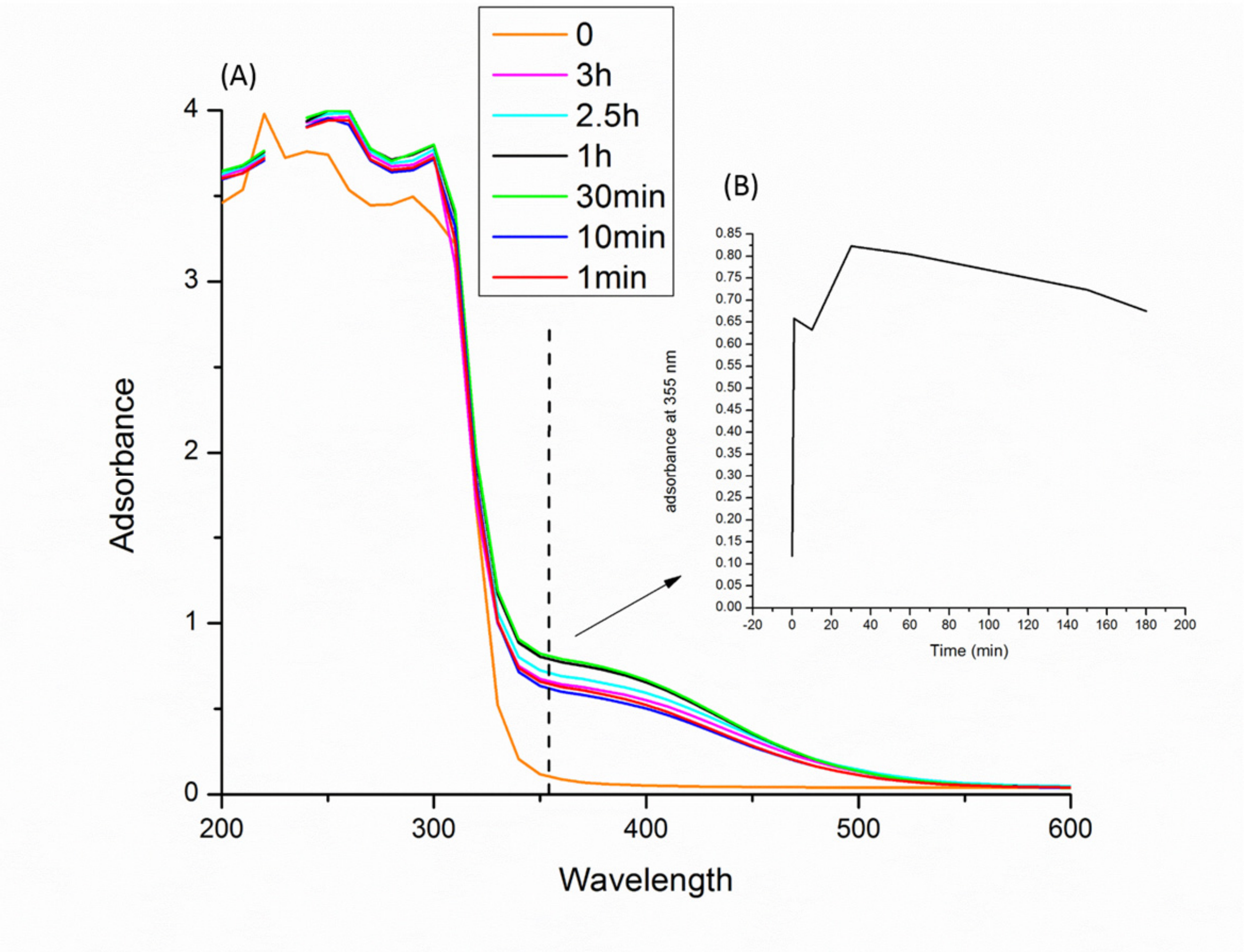

Figure S6. Time dependence of UV-vis absorption of TiO_2-DA particle suspensions. UV-vis absorption spectra of DA-modified TiO_2 with (A) different incubation times and (B) its UV absorbance kinetic curve at a wavelength of 355 nm.

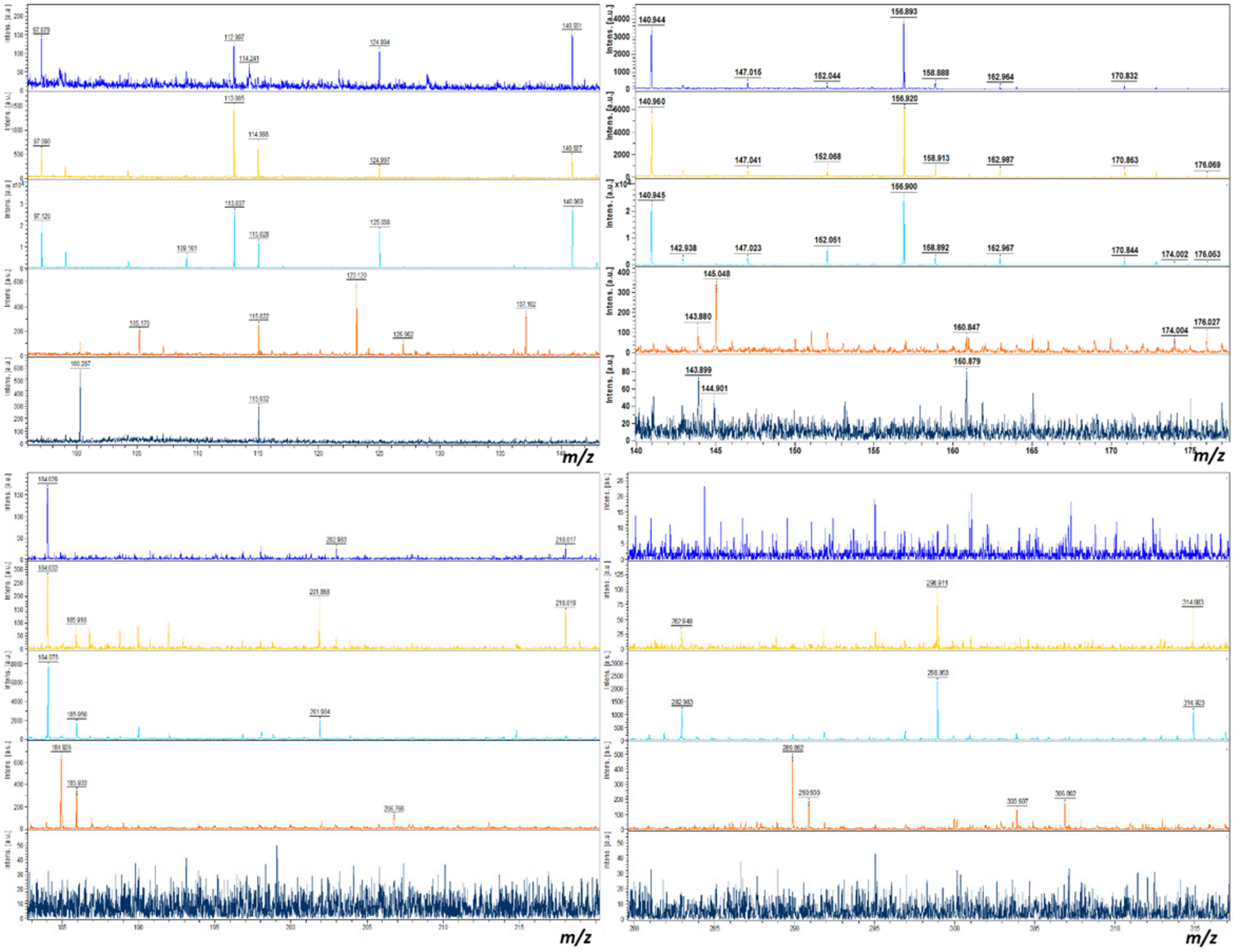

Figure S7. Comparison of mass spectra from mouse brain tissue obtained using (blue) TiO_2 sub-micron particle-, (yellow) TiO_2-DA sub-micron particle-, and (light blue) TiO_2-DA monolith-assisted LDI, and mass spectra of blank samples with (dark blue) TiO_2 sub-micron particle- and (orange) TiO_2-DA monolith-assisted LDI.

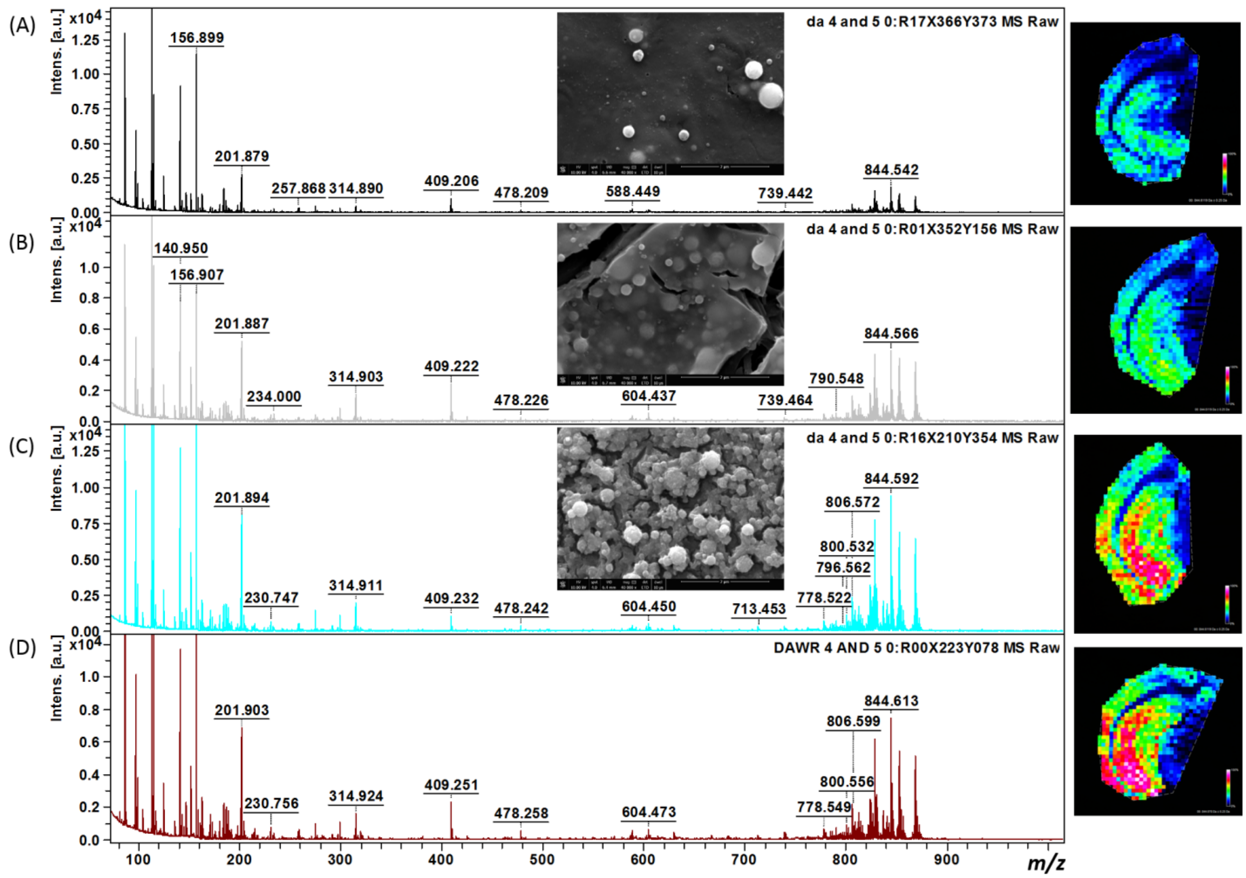

Figure S8. Effect of water content in reaction solutions for further hydrolysis/condensation on TiO_2-DA structure morphology and TiO_2-DA materials-assisted LDI MSI measurements. (Left) Representative mass spectra acquired from mouse brain hemisphere sections coated with TiO2-DA materials generated in the presence of (A) 1%, (B) 2.5%, (C) 5%, and (D) 10% water in ethanol solution (reaction time is 60 min each). (Insets) SEM images of corresponding samples coated with TiO2-DA materials. (Right) Ion maps of representative lipid (*m/z* 844.5) distributions in the sections. Acquisitions were performed in the low spatial resolution mode with a 100-µm raster step size using settings producing a laser footprint ~100-µm in diameter.

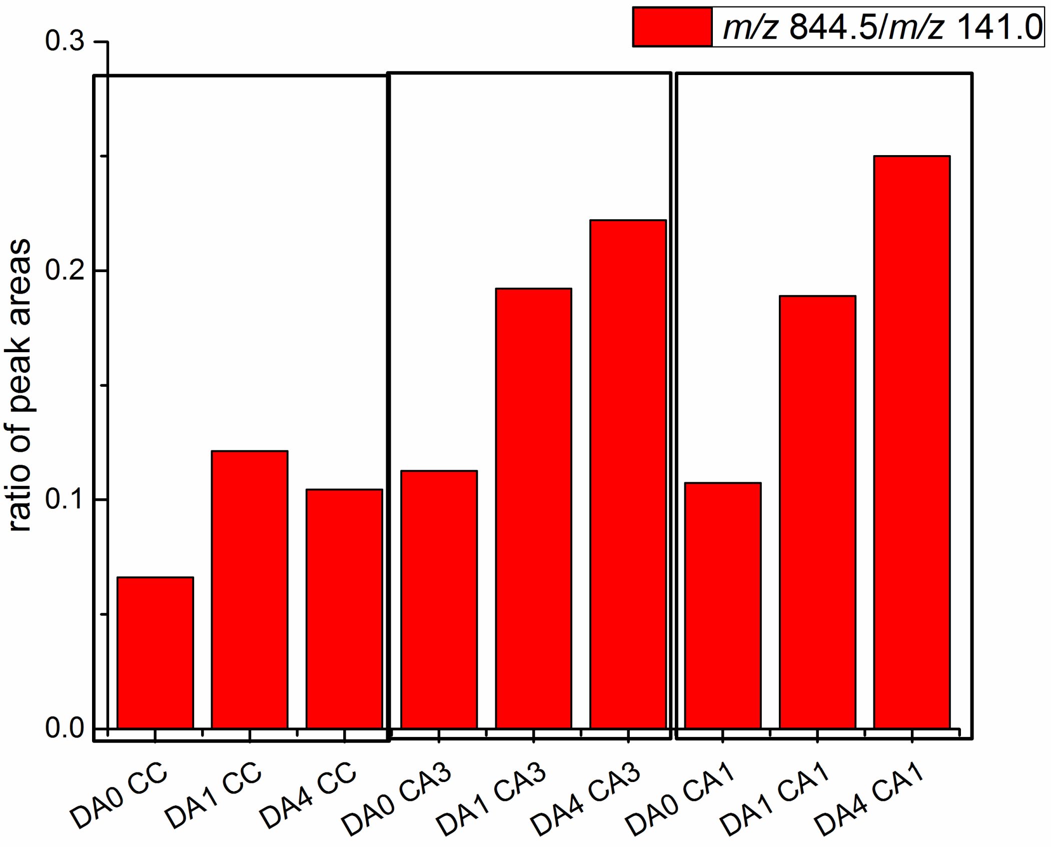

Figure S9. Ratios of an intact lipid to its fragment peak areas as determined using different TiO_2-based LDI MS methods. The intact lipid (*m/z* 844.5) to fragment (*m/z* 141.0) ratios were calculated using data acquired from different subregions of mouse brain using LDI MS assisted with TiO_2 sub-micron particles (DA0), TiO_2-DA sub-micron particles (DA1), and TiO_2-DA monoliths (DA4). CA1, region I of hippocampus proper; CA3, region III of hippocampus proper; CC, corpus callosum.

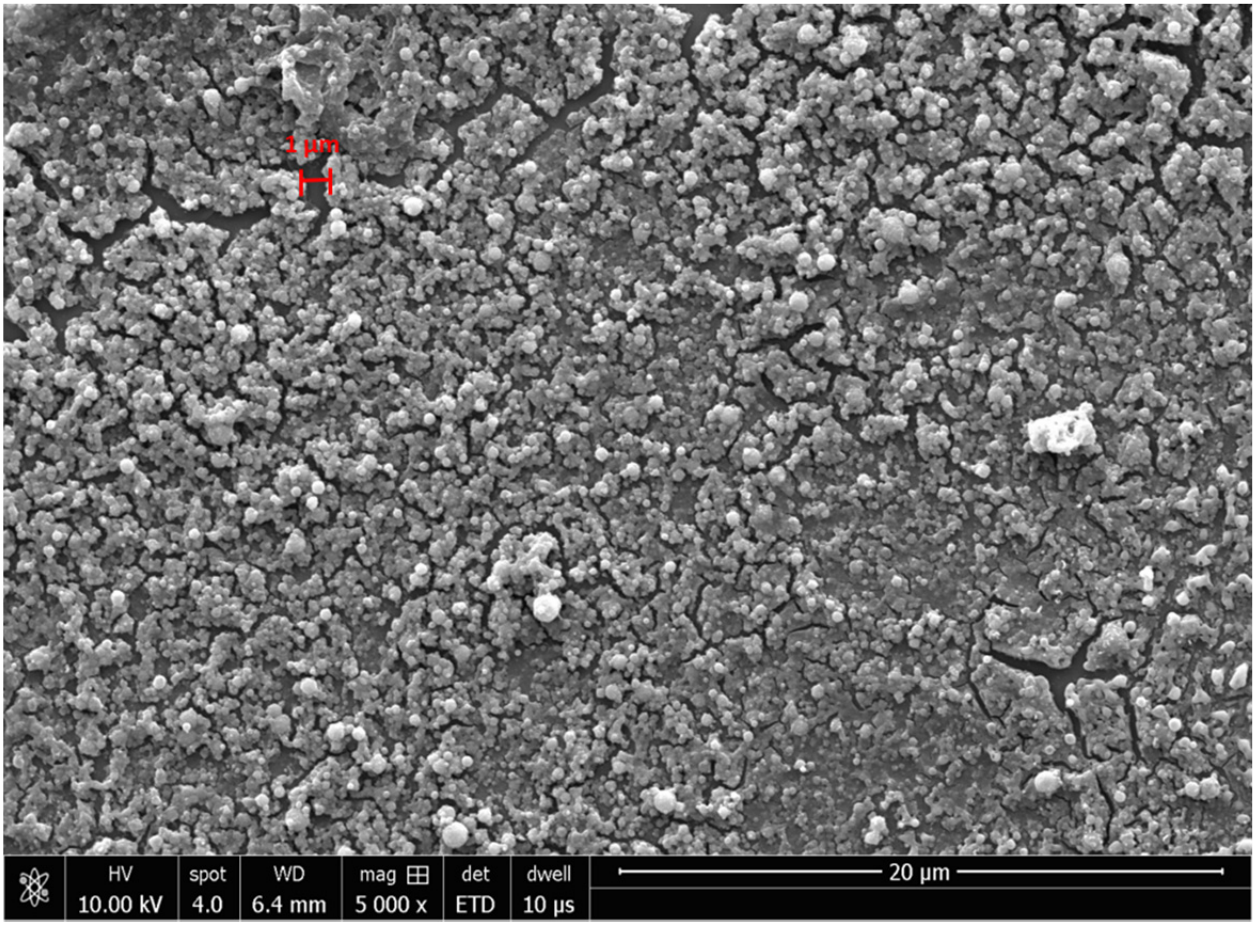

Figure S10. Scanning electron microscopy image of the TiO2-DA monolith surface with low magnification (×5000).

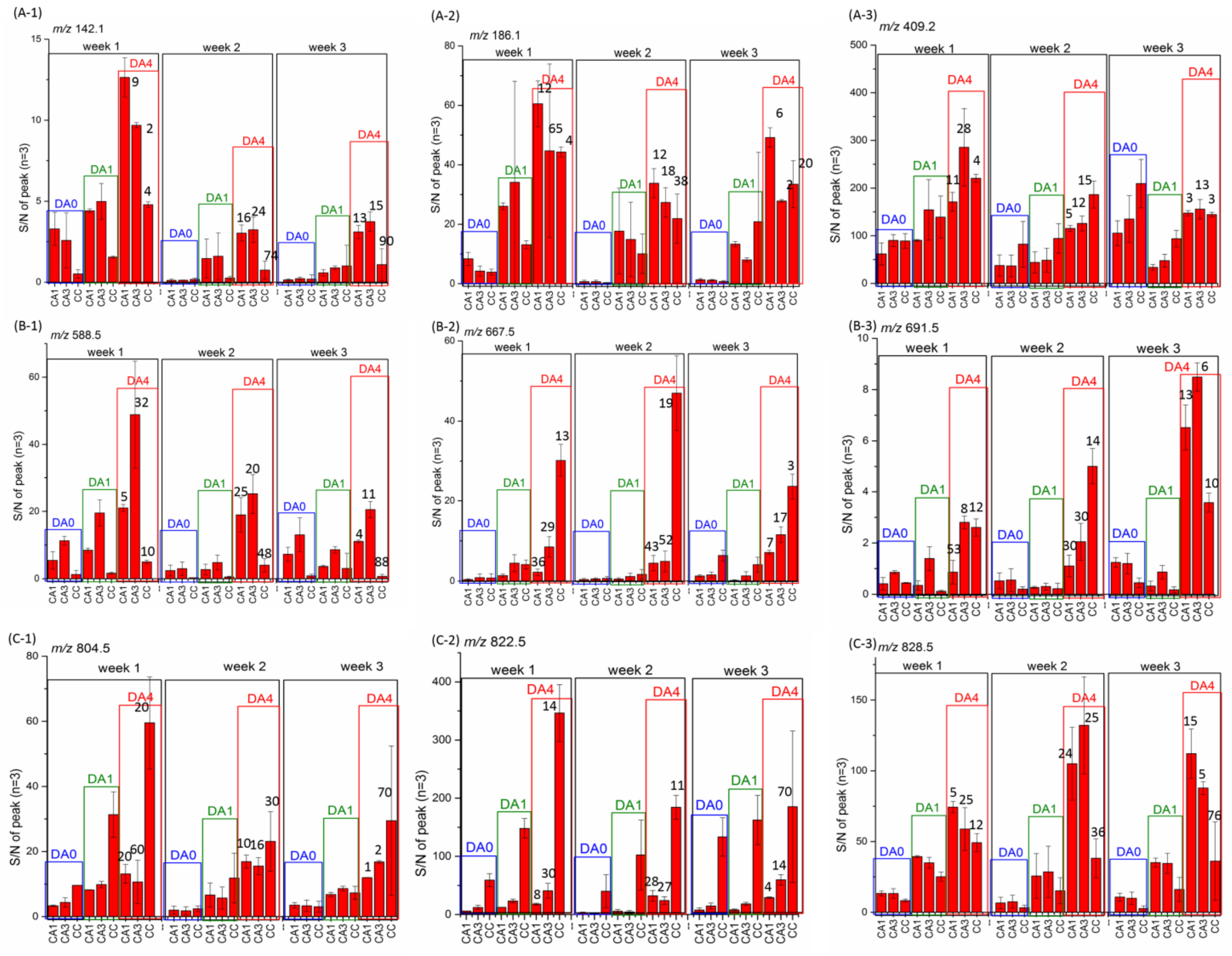

Figure S11. Repeatability of the unmodified TiO_2 sub-micron particle (DA0)-, TiO_2-DA sub-micron particle (DA1)-, and TiO_2-DA monolith (DA4)-assisted LDI MS measurements. Histograms depict average peak S/Ns of different molecular signals: (A) *m/z* 100–500, (B) *m/z* 500–700, and (C) *m/z* 700–900, acquired from region I of hippocampus (CA1), region III of hippocampus (CA3), and corpus callosum (CC) of different animals prepared and analyzed in different weeks. Average peak S/Ns with standard deviation error bars calculated using data obtained from measurements of adjacent brain slice triplicates collected from the same animals. The slices were deposited on different ITO glass slides and coated with the TiO_2 materials. The relative standard deviations of the triplicate data acquired using TiO_2-DA monoliths are labeled on the corresponding bar graphs. Columns are positioned and boxed according to the week when samples were collected and analyzed.

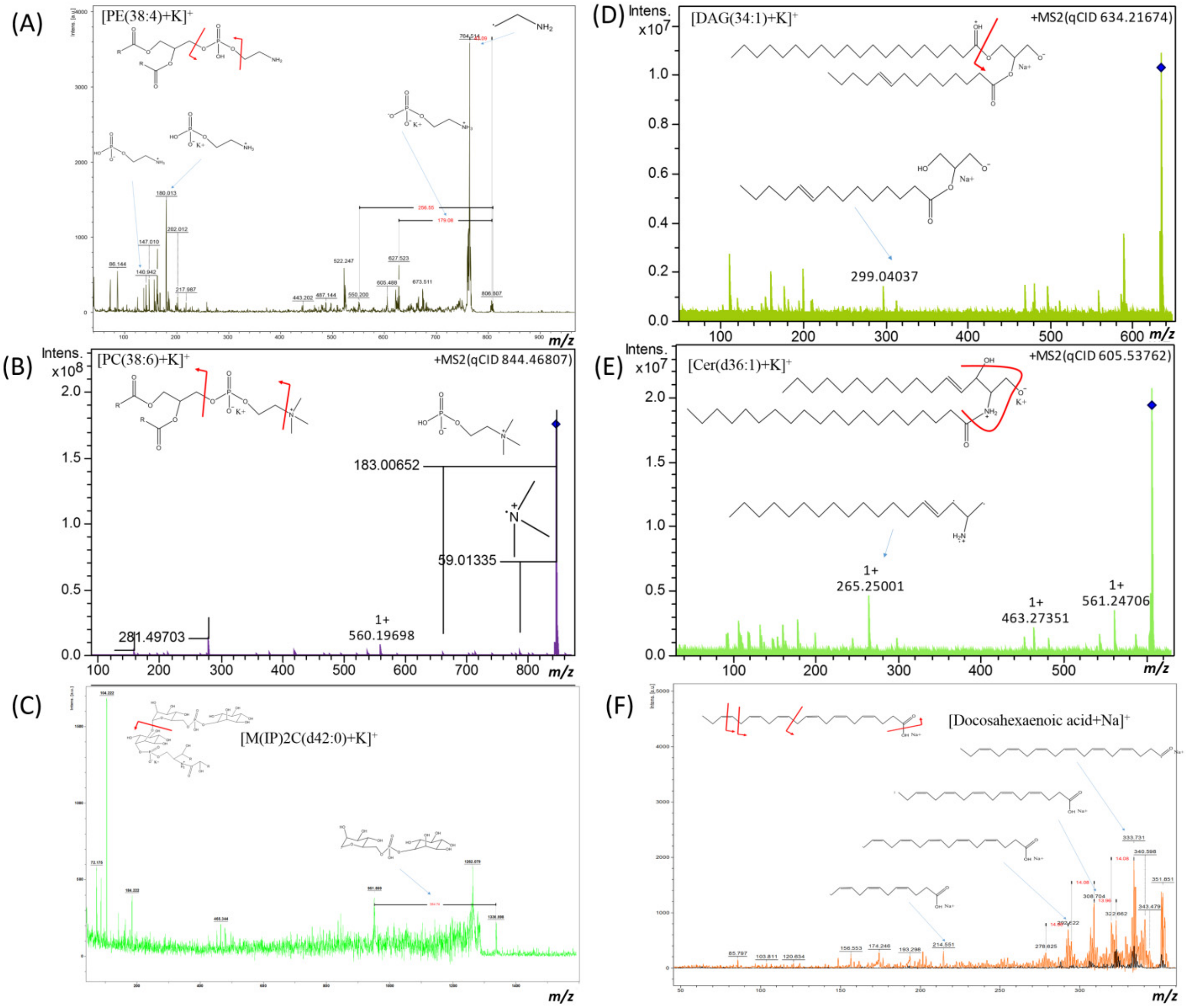

(A)
[PE(38:4)+K]+
m/z
(B)
Intens. x10^8
[PC(38:6)+K]+
+MS2(qCID 844.46807)
183.00652
59.01335
281.49703
1+ 560.19698
m/z
(C)
[M(IP)2C(d42:0)+K]+
m/z
(D)
Intens. x10^7
[DAG(34:1)+K]+
+MS2(qCID 634.21674)
299.04037
m/z
(E)
Intens. x10^7
[Cer(d36:1)+K]+
+MS2(qCID 605.53762)
1+ 265.25001
1+ 463.27351
1+ 561.24706
m/z
(F)
[Docosahexaenoic acid+Na]+
m/z

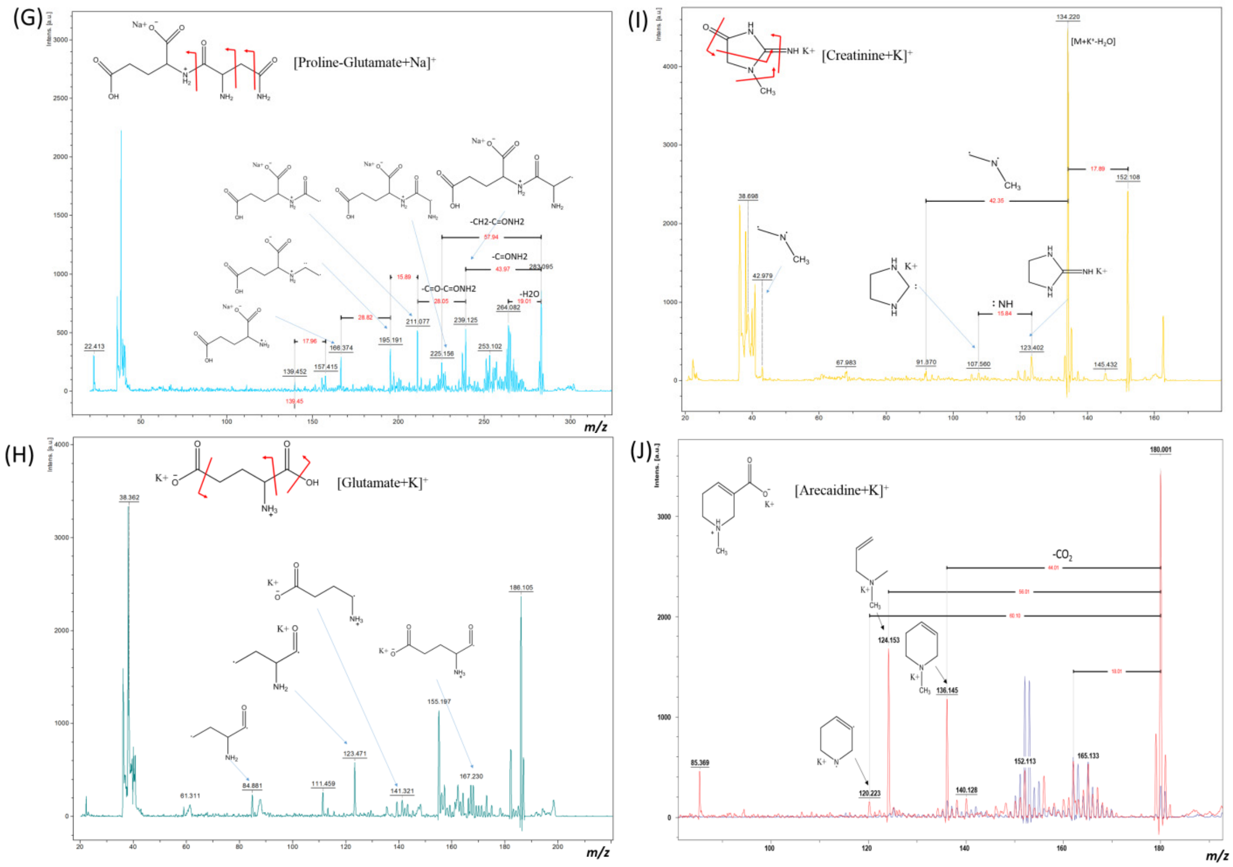

Figure S12. MS/MS spectra of different endogenous compounds detected in mouse brain tissues during TiO_2-DA monolith-assisted LDI MSI experiments. (A) PE, phosphatidylethanolamine; (B) PC, phosphatidylcholine; (C) M(IP)2C, mannose-(inositol-P)2-ceramide; (D) DAG, diacylglycerol; (E) Cer, ceramide; (F) docosahexaenoic acid; (G) proline-glutamate; (H) glutamate; (I) creatinine; (J) arecaidine.

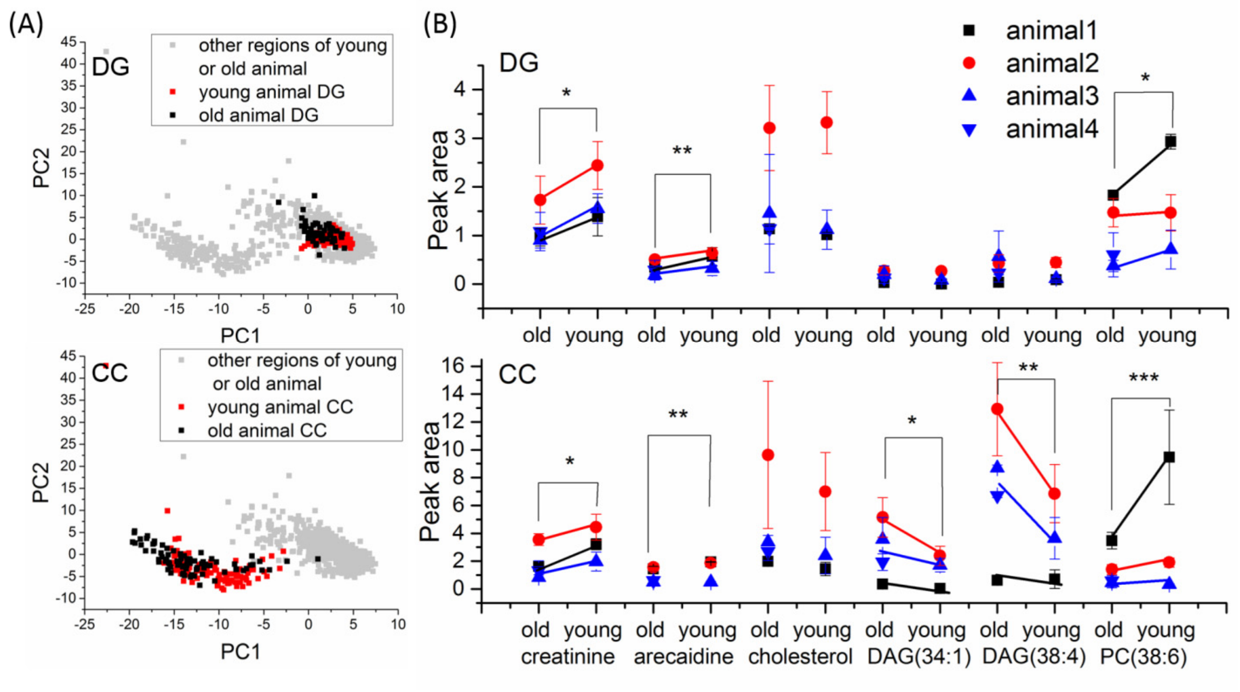

Figure S13. Results of PCA and ANOVA statistical analyses of data acquired from old and young mice samples. (A) PCA score plots of the TiO_2-DA monolith-assisted LDI MSI dataset acquired from different cerebrum regions of young and old mice. (B) Statistical comparison of average peak areas of different identified molecules detected in brain samples of old and young mice. p values were calculated using two-way ANOVA for data sets representing different age groups (see details in Table S4). DG, dentate gyrus; CC, corpus callosum. ***, p value<0.001, **, p value<0.01, *, p value<0.05.

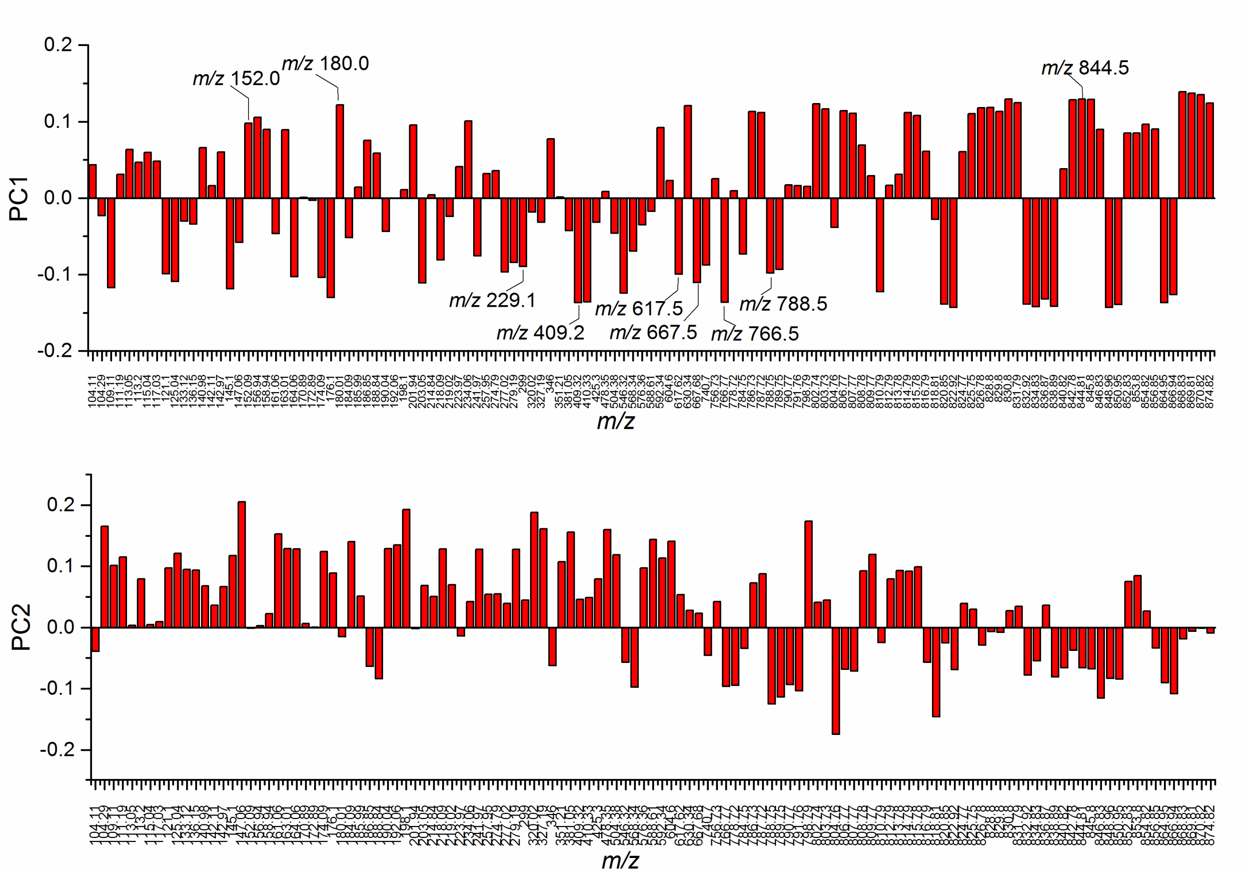

Figure S14. Loading plots of (top) PC1 and (bottom) PC2 of data collected from young and old mice samples using the optimized TiO_2-DA-assisted LDI method.

Table S1. Tukey test results for all of the data sets presented in Figure S1 and Figure S3.

Figure	X axis of two datasets	P values of two data sets determined by Tukey test					
		m/z 162.9	*m/z* 140.9	*m/z* 136.1	*m/z* 868.5	*m/z* 852.5	*m/z* 806.5
S1B	10 min–0 min	0.06076	0.0074	0.00674	0.5422	0.0285	0.99981
	3 h–0 min	0.05081	0.00253	0.00719	0.34371	0.15104	0.99797
	3 h–10 min	0.99961	0.50564	0.99997	0.98635	0.48654	0.99991
	12 h–0 min	0.19164	0.0024	0.00686	0.00151	1.62E-04	0.05736
	12 h–10 min	0.78256	0.46468	1	0.00378	0.00106	0.06669
	12 h–3 h	0.689	0.99998	0.99999	0.00511	4.82E-04	0.0757
	24 h–0 min	0.09771	0.00319	0.00637	0.0021	1.43E-04	0.0574
	24 h–10 min	0.98556	0.70089	0.99999	0.00559	8.84E-04	0.06674
	24 h–3 h	0.95422	0.99314	0.99969	0.00773	4.15E-04	0.07576
	24 h–12 h	0.95818	0.98526	0.99996	0.96667	0.99456	1
S3A	0.0025–0 (concentration of HNO_3, M)	0.99775	0.01929	0.81613	0.00272	0.12439	0.0153
	0.005–0	0.81737	0.02544	0.95645	5.36E-04	0.02467	0.00343
	0.005–0.0025	0.73214	0.97331	0.57233	0.02932	0.31235	0.13918
	0.01–0	0.47693	0.0052	0.6885	1.55E-04	0.01501	0.00131
	0.01–0.0025	0.40543	0.23175	0.31588	0.00199	0.15026	0.0201
	0.01–0.005	0.89054	0.15635	0.91174	0.02238	0.86676	0.20685
S3B	0.005–0 (concentration of H_3PO_4, M)	0.95288	0.18956	0.29412	0.33198	0.38204	0.27629
	0.01–0	0.08448	0.0992	0.09113	0.03231	0.05654	0.07972
	0.01–0.005	0.10514	0.7225	0.43322	0.09655	0.18038	0.38979

Table S2. Peak list collected from averaged mass spectra acquired from mouse brain tissues and corresponding blanks using TiO_2 sub-micron particle-, TiO2-DA sub-micron particle-, and TiO2-DA monolith-assisted LDI MS.

	**Averaged normalized peak area (mass spectra acquired from the corpus callosum)**					**Blank sample (background signal of TiO_2 materials)**	
m/z	TiO_2 sub-micron particles	TiO_2-DA sub-micron particles	TiO_2-DA monolith	Analyte identity	Molecular mass error (Δppm)	TiO_2 sub-micron particles	TiO_2-DA sub-micron particles/monolith
104.26	1.135	1.642	1.668				
109.08	3.944	4.231	7.517				
111.15	1.954	0.742	0.325				
113.02	5.818	20.998	51.533				
115.01	1.905	8.061	22.988			*	*
117.01	0.358	0.899	2.216				
121.07	1.362	0.928	1.525				
123.12	-		-				*
125.01	11.659	10.706	20.918				
126.962	-		-				*
127.01	0.735	0.577	0.912				
136.11	0.365	0.689	1.799				
137.102	-		-				*
138.96	0.502	0.382	0.668				
140.95	12.574	15.461	35.571				
142.08	0.345	0.216	0.339				
142.94	1.615	1.809	3.709				
143.895	-		-			*	*
145.07	-	3.781	4.521			*	*
147.03	2.099	2.692	4.223				
152.05	0.327	0.820	2.905				
154.05	0.245	0.191	0.332				
154.9	0.434	0.614	1.362				
156.9	6.866	9.316	26.446				
158.9	1.531	1.700	3.949				
161.01	0.521	1.300	1.356				
162.97	0.744	0.978	1.854				
164.01	0.766	0.736	0.773				*
167.02	0.203	0.083	0.029				
167.98	0.270	0.435	0.622				
168.95	0.312	0.330	0.522				
169.98	0.241	0.508	0.947				*
170.84	0.469	0.873	2.294				
172.02	0.166	0.360	0.557				

172.84	0.504	0.882	2.202				
174.05318 [a]	-	0.668	1.272	Dopamine quinone+Na	3		*
176.06866 [a]	-	2.225	2.756	Dopamine +Na	2		*
179.97	0.587	0.812	0.844				
184.04	7.996	6.440	5.110				
184.928	-	-	-				*
185.94	0.294	0.920	2.150				*
186.8	0.342	0.573	1.246				
188.8	0.310	0.451	1.027				
189.98	0.270	0.735	1.122				*
192	-	0.832	0.859				
195.95	0.141	0.112	0.143				
198.03	0.710	0.843	1.250				
201.89 [a]	-	0.851	2.969	2-Thiothiazolidine-4-carboxylic acid+K	24		
202.99	1.136	0.763	0.546				
206.766	-	-	-				*
214.78	0.526	0.543	1.259				
215.88	0.163	0.143	0.216				
218.02	1.600	0.773	0.418				
218.96	0.771	0.547	0.295				
223.717	-	-	-				*
224.89	0.087	0.161	0.166				
233.99	0.658	0.449	0.201				
240.91	0.289	0.353	0.369				
241.91	0.299	0.423	0.598				
257.88	0.238	0.314	0.534				
258.77	0.709	0.783	2.059				
260.97	1.216	1.046	1.539				
263.92	0.577	0.401	0.489				
264.8	-	-	-				*
274.72	0.501	0.391	1.022				
279.1	0.549	0.348	0.165				
280.92	0.292	0.349	0.865				
282.95	0.562	1.024	2.270	Glutamyl-Asparagine+Na	21		
289.892	-	-	-				*
292.86	0.242	0.189	0.135				
294.9	0.232	0.253	0.279				
296.91	0.314	0.417	0.898				
298.92 [a]	-	1.288	2.787	Glutamyl-Asparagine+K	27		

303.907	-	-	-				*
305.13	0.366	0.293	0.278				
306.892	-	-	-				*
307.13	0.443	0.397	0.203				
314.9	0.280	0.696	1.487				
320.9	0.305	0.211	0.226				
322.87	0.150	0.155	0.175				
327.07	0.295	0.405	0.569				
335.11	0.289	0.250	0.169				
336.95	0.217	0.199	0.141				
340.88	0.140	0.069	0.004				
343.06	0.145	0.306	0.214				
351.08	0.343	0.259	0.367				
355.14	0.165	0.213	0.139				
363.14	0.287	0.150	0.080				
367.06	0.242	0.125	0.047				
369.83	-	-	-				*
393.175	-	-	-				*
407.18	1.696	0.360	0.582				
409.21	15.746	6.786	7.994				
410.21	4.020	1.577	1.928				
413.13	0.094	0.042	0.027				
415.11	0.178	0.067	0.089				
417.13	0.178	0.065	0.018				
425.18	1.090	0.553	0.617				
429.12	0.126	0.065	0.019				
431.2	0.171	0.045	0.058				
439.18	0.253	0.093	0.051				
441.12	0.494	0.243	0.190				
453.08	0.254	0.094	0.078				
457.19	0.521	0.148	0.057				
459.14	0.210	0.071	0.006				
465.13	0.259	0.198	0.195				
469.02	0.189	0.092	0.078				
481.11	0.116	0.067	0.085				
495.19	0.118	0.043	0.042				
497.07	0.126	0.105	0.052				
502.17	0.203	0.112	0.065				
551.25	0.151	0.098	0.089				
564.16	0.121	0.107	0.055				
586.45	0.277	0.144	0.110				

588.46	0.439	0.339	0.346				
592.18	0.272	0.283	0.220				
604.43	0.101	0.079	0.064				
617.43	0.616	0.270	1.650				
630.18	0.109	0.156	0.037				
633.42	0.200	0.115	0.245				
655.4	0.233	0.152	0.238				
667.52	1.483	0.897	4.515				
683.46	0.393	0.259	0.562				
691.48	0.205	0.140	0.364				
697.45	0.176	0.128	0.137				
701.38	0.178	0.160	0.127				
707.47	0.173	0.103	0.114				
711.46	0.198	0.140	0.122				
713.42	0.174	0.141	0.218				
723.51	0.527	0.297	0.201				
740.47	0.444	0.416	0.260				
766.61	2.754	2.969	1.924				
772.54	0.388	0.710	0.831				
774.58	0.250	0.410	0.490				
778.49	0.764	1.014	1.120				
782.58	0.942	1.350	0.946				
784.55	0.588	0.775	0.527				
788.57	0.690	1.747	2.049				
790.56	0.607	1.604	1.797				
792.57	0.695	0.748	0.772				
794.67	2.612	2.330	1.173				
802.53	0.322	0.523	0.546				
804.59	0.520	1.319	1.485				
806.6	1.311	2.246	3.058				
807.55	0.357	0.872	1.214				
808.58	0.813	1.076	1.521				
809.49	0.770	1.000	1.013				
810.57	0.974	1.602	1.269				
811.6	0.715	0.833	0.601				
812.58	0.804	1.405	1.595				
814.55	0.469	0.831	0.752				
816.57	0.501	1.028	0.956				
818.62	0.609	0.953	0.710				
820.69	1.250	1.191	0.326				
822.72	20.355	22.901	16.706				

824.55	3.655	4.443	3.970				
826.49	0.518	0.839	0.892				
828.59	0.368	1.286	2.329				
830.55	0.776	1.178	1.030				
832.75	5.830	11.035	7.467				
834.65	0.656	1.412	0.877				
836.73	11.053	13.412	10.642				
837.58	3.880	5.202	4.485				
838.67	7.797	10.591	8.178				
840.53	1.806	1.984	0.950				
844.55	0.375	1.110	1.871				
846.57	0.306	0.341	0.472				
848.73	8.926	16.396	11.044				
849.73	2.612	5.512	3.708				
850.73	7.836	10.570	7.178				
851.74	3.461	4.427	2.865				
852.59	4.499	6.522	6.640				
856.59	0.086	0.152	0.211				
864.7	2.824	4.656	3.450				
865.72	0.954	1.687	1.185				
866.72	3.205	4.463	3.413				
867.75	0.749	1.285	0.884				
868.6	0.689	0.919	1.094				
884.61	0.179	0.149	0.167				
1268.44	0.093	0.095	0.058				
1336.53	0.092	0.089	0.034				

* Peak is observed in this *m/z*.

[a] Peak identities are assigned using the precise molecular masses determined by FTICR MS.

Table S3. Tukey test results for all of the data points presented in Figure 2.

X axis of two data sets		P values of two data sets by Tukey test								
		m/z 152.1	*m/z* 588.5	*m/z* 298.9	*m/z* 822.5	*m/z* 409.2	*m/z* 828.5	*m/z* 667.5	*m/z* 804.5	*m/z* 136.1
CA1	DA1-DA0	0.2818	1	0.9176	1	1	0.9049	0.9851	0.9285	0.9981
	DA4-DA0	0.0002	0.00005	0.002	0.8236	0.0457	0.00005	0.5138	0.0168	0.9957
	DA4-DA1	0.0342	0.00007	0.0272	0.896	0.0807	0.0007	0.0128	0.1885	0.4655
CA3	DA4-DA0	0.0011	0.000009	0.00004	0.9555	0.02	0.000002	0.456	0.0322	0.8943
	DA4-DA1	0.06	0.000003	0.001	0.9782	0.0481	0.00003	0.5973	0.2278	0.9995
	DA1-DA0	0.5885	0.9968	0.7131	1	0.9997	0.85	0.9951	0.9728	0.2956
CC	DA1-DA0	0.91	1	0.1188	0.0831	0.9996	0.9938	1	0.2639	0.6837
	DA4-DA0	0.1057	0.8239	0.00002	0.00002	0.004	0.3171	0.00000005	0.0006	0.1856
	DA4-DA1	0.6924	0.8657	0.004	0.0105	0.0125	0.7871	0.0000006	0.1171	0.4367

Abbreviations: CA1, region I of hippocampus proper; CA3, region III of hippocampus proper; CC, corpus callosum. TiO_2 sub-micron particles, (DA0); TiO_2-DA sub-micron particles, (DA1); and TiO_2-DA monoliths (DA4).

Table S4. List of identified or putatively identified analytes detected in mouse brain using TiO_2-DA monolith-assisted LDI MS, DHB-assisted MALDI MS, and 9-AA assisted MALDI MS. Precise *m/z* and mass errors (Δppm) of measurements are reported. Due to the use of precise molecular masses and/or molecular fragmentation patterns for characterization of the listed analytes, different levels of confidence in their identification are stated (see footnotes for the table).

Mass to charge ratio (*m/z*)	Mass error (Δppm)	Compounds	Detected ions	Analyte chemical class	Ref.
**TiO_2-DA-assisted LDI MS**					
170.03272	0.7	Creatine	$[M+K]^+$	Alkaloid	[3-5]
172.037	0.6	5,6-dihydroxyindole	$[M+Na]^+$	Alkaloid	[6,7]c
180.0422 [a]	0.5	Arecaidine	$[M+K]^+$	Alkaloid	[8]c
295.07237	0.2	5-S-Cysteinyldopamine	$[M+Na]^+$	Alkaloid	[9,10]c
136.04855	3	Creatinine	$[M+Na]^+$	Alkaloid	[11]c
152.02295[a]	5	Creatinine	$[M+K]^+$	Alkaloid	[11]c
216.042[a]	0.8	5-Hydroxytryptophol	[M+K]+	Alkaloid	[12]c
225.20538 [a]	1	Spermine	$[M+Na]^+$	Alkaloid	[3,13]
241.17888 [a]	3	Spermine	$[M+K]^+$	Alkaloid	
139.08317[a]	7	Aminopentanamide	$[M+Na]^+$	Amino acids	[14] d
142.02682	2	Aminobutanoic acid	$[M+K]^+$	Amino acids	[3,15]
154.02049	38	Proline	$[M+K]^+$	Amino acids	[16,17]
167.0219	1	2-amino-4-cyano-butanoic acid	$[M+K]^+$	Amino acids	[18-21]
168.0057 [a]	0.6	Pyroglutamic acid	$[M+K]^+$	Amino acids	[21,22]c
169.0585	0.6	Glutamine	$[M+Na]^+$	Amino acids	[16,23]
186.01661 [a]	1	Glutamate	$[M+K]^+$	Amino acids	[16]
196.00076	0.3	Amino-muconic acid	$[M+K]^+$	Amino acids	[24]d
198.08906	0.2	Amino-octanoic acid	$[M+K]^+$	Amino acids	[25] d
234.07355 [a]	0.6	Methoxytyrosine	$[M+Na]^+$	Amino acids	[15]
586.4919	42	Cer(d36:2)	$[M+K]^+$	Cer	[26]
588.53436 [a]	2	Cer(d36:1)	$[M+Na]^+$	Cer	[26]
604.5066	7	Cer(d36:1)	$[M+K]^+$	Cer	[26]
429.23852	3	Hydroxy-oxo-cholan-oic Acid	$[M+K]^+$	CL	[27,28]c
439.29546	4	OH-7-Dehydrocholesterol	$[M+K]^+$	CL	[29]
465.33124	5	Cholesta-6,8(14)-dien-3beta,5alpha-diol	$[M+K]^+$	CL	d
691.58617 [a]	19	18:0 Cholesteryl ester	$[M+K]^+$	CL	[30,31]
409.34237 [a]	4	Cholesterol	$[M+Na]^+$	CL	[32-36]
425.31629	5	Cholesterol	$[M+K]^+$	CL	
667.53166	10	DAG(38:4)	$[M+Na]^+$	DAG	[37]c
683.49536 [a]	8	DAG(38:4)	$[M+K]^+$	DAG	
711.5751 [a]	20	DAG(41:3)	$[M+Na]^+$	DAG	d
617.51259 [a]	7	DAG(34:1)	$[M+Na]^+$	DAG	[38]
633.48768	3	DAG(34:1)	$[M+K]^+$	DAG	
655.46536 [a]	6	DAG(36:4)	$[M+K]^+$	DAG	d
259.05220	10	Met-Ala	$[M+K]^+$	Di-peptides	[39]c
277.0617	1	Gly-Tyr	$[M+Na]^+$	Di-peptides	[40]c
283.06917	0.2	Pro-Glu	$[M+K]^+$	Di-peptides	[39]c

299.04309 [a]	27	Glu-Asn	$[M+K]^+$	Di-peptides	[41]c
279.22797	1	Palmitic acid	$[M+Na]^+$	FA	[42]
293.18727	1	FA16:1	$[M+K]^+$	FA	[43]d
295.20288	1	FA14:0	$[M+K]^+$	FA	d
337.21264	3	Hydroxy-oleic acid	$[M+K]^+$	FA	[44]c
341.1872	1	FA20:5	$[M+K]^+$	FA	[45]
355.25968	2	Docosatetraenoic acid (22:4)	$[M+Na]^+$	FA	[46-48]c
363.2657	0.8	FA21:1	$[M+K]^+$	FA	d
551.4999	36	FA36:4	$[M+Na]^+$	FA	d
305.244 [a]	3	FA18:1	$[M+Na]^+$	FA	[35,45]
321.2177	4	FA18:1	$[M+K]^+$	FA	
307.26019	1	Stearic acid(fa18:0)	$[M+Na]^+$	FA	[35]
323.23377	2	Stearic acid(fa18:0)	$[M+K]^+$	FA	
327.22878 [a]	2	Arachidonic acid	$[M+Na]^+$	FA	[34,45]
343.20437	2	Arachidonic acid	$[M+K]^+$	FA	
351.22846 [a]	3	Docosahexaenoic acid	$[M+Na]^+$	FA	[34]
367.202	3	Docosahexaenoic acid	$[M+K]^+$	FA	
774.59036	6	GalCer(d38:3)	$[M+Na]^+$	GlcCer	d
810.58796	2	GlcCer(d38:1)	$[M+K]^+$	GlcCer	d
848.62954	9	GlcCer(d42:2)	$[M+K]^+$	GlcCer	[49]
1268.718 [b]	69	M(IP)2C(d36:0)	$[M+K]^+$	M(IP)2C	[50]c
1336.818 [b]	82	M(IP)2C(d42:0)	$[M+K]^+$	M(IP)2C	[50]c
417.23857	2	PA(P-16:0)	$[M+Na]^+$	PA	[51]d
441.23772	0.3	CPA(18:1)	$[M+Na]^+$	PA	[52]d
701.44678	7	PA(33:0)	$[M+K]^+$	PA	d
707.49624	3	PA(O-36:3)	$[M+Na]^+$	PA	d
713.44667	7	PA(34:1)	$[M+K]^+$	PA	[53]
723.48923	5	PA(36:2)	$[M+Na]^+$	PA	[54]
415.22086	2	PA(16:0)	$[M+Na]^+$	PA	d
431.1935	5	PA(16:0)	$[M+K]^+$	PA	
782.56108	7	PC(34:1)	$[M+Na]^+$	PC	[49]
826.56495	8	PC(36:1)	$[M+K]^+$	PC	[49]
844.46889 [a]	66	PC(38:6)	$[M+K]^+$	PC	[55]
864.62704	3	PC(P-41:2)	$[M+K]^+$	PC	[49]d
866.64113	1	PC(O-40:2)	$[M+K]^+$	PC	[49]d
784.5217 [a]	4	PC(33:1)	$[M+K]^+$	PC	[49]d
832.66687	4	PC(38:4)	$[M+Na]^+$	PC	[55]
848.55262	4	PC(38:4)	$[M+K]^+$		
740.47886 [a]	1	PE(34:1)	$[M+K]^+$	PE	[55]
772.52012	6	PE(35:0)	$[M+K]^+$	PE	d
778.47591 [a]	3	PE(36:4)	$[M+K]^+$	PE	[56]c
790.52275 [a]	10	PE(p-38:4)	$[M+K]^+$	PE	[55]
802.47473	4	PE(38:6)	$[M+K]^+$	PE	[56]c
804.4907 [a]	4	PE(38:3)	$[M+K]^+$	PE	[57]c
806.50434 [a]	6	PE(38:4)	$[M+K]^+$	PE	[54]
814.51051	5	PE(P-40:6)	$[M+K]^+$	PE	d
828.48788 [a]	7	PE(40:7)	$[M+K]^+$	PE	[58]c

830.50526	5	PE(40:6)	$[M+K]^+$	PE	[59]
834.53751	4	PE(40:4)	$[M+K]^+$	PE	[54]
836.66568 [a]	13	PE(42:9)	$[M+Na]^+$	PE	d
852.48229	13	PE(42:9)	$[M+K]^+$	PE	
884.49514	69	PE(44:7)	$[M+K]^+$	PE	d
782.5631 [a]	21	PE(O-37:2)	$[M+K]^+$	PE	d
794.61321 [a]	5	PE(P-39:1)	$[M+Na]^+$		d
810.58796	2	PE(P-39:1)	$[M+K]^+$	PE	
824.46241 [a]	36	PE(40:9)	$[M+Na]^+$	PE	d
822.64786	60	PE(40:2)	$[M+Na]^+$		d
838.6233 [a]	60	PE(40:2)	$[M+K]^+$	PE	
697.47287	6	PE-Cer(d34:2)	$[M+K]^+$	Cer-PE	d
413.12415	3	Cys Gly Pro Val	$[M+K]^+$	Peptides	e
453.17582	2	Gly Glu Pro Ile	$[M+K]^+$	Peptides	
457.20921	0.2	Met Leu Ala Thr	$[M+Na]^+$	Peptides	
459.22234	2	Thr Leu Gly Phe	$[M+Na]^+$	Peptides	
469.14905	1	Glu Trp Pro	$[M+K]^+$	Peptides	
481.20686	1	Asp Val Leu Pro	$[M+K]^+$	Peptides	
495.1643	0.6	Gly Phe Ala Tyr	$[M+K]^+$	Peptides	
497.17951	1	Asn Asn Pro Met	$[M+Na]^+$	Peptides	
502.2659	4	Ser Phe Val Lys	$[M+Na]^+$	Peptides	
564.2442	2	Val Tyr Asn Phe	$[M+Na]^+$	Peptides	
592.23942	2	Phe Glu Phe Gln	$[M+Na]^+$	Peptides	
630.19389	6	Phe Cys Trp His	$[M+K]^+$	Peptides	
**DHB-assisted MALDI MS**					
606.848	19	Cer(d39:2)	$[M+H]^+$	Cer	
369.35762	13	5beta-Cholestane-3alpha,12alpha-diol	$[M+H-2H_2O]^+$	CL	
391.29314/390.887	2	(23S)-3α,7β,23-Trihydroxy-5β-cholan-24-oic Acid	$[M+H-H_2O]^+$	CL	
551.51348	1	12:0 Cholesteryl ester	[M+H-H2O]+	CL	
577.52959	9	14:1 Cholesteryl ester	[M+H-H2O]+	CL	
790.55310	7	GalCer(d38:3)	$[M+K]^+$	GalCer	
958.59544 a	9	MIPC(t34:0(2OH))	$[M+H-H_2O]^+$	MIPC	
734.58424	16	PC(32:0)	[M+H]+	PC	[49]
756.56601	21	PC(34:3)	$[M+H]^+$	PC	[55]
760.6013 [a]	20	PC(34:1)	$[M+H]^+$	PC	[49]
762.61858 a	14	PC(34:0)	$[M+H]^+$	PC	[55]
782.58283	19	PC(36:4)	$[M+H]^+$	PC	[55]
784.59648 [a]	21	PC(36:3)	$[M+H]^+$	PC	[55]
788.63513 [a]	2	PC(O-37: 1)	$[M+H]^+$	PC	
804.56972	20	PC(38:7)	$[M+H]^+$	PC	[55]
806.52966 [a]	23	PC(P-36:3)	$[M+K]^+$	PC	
808.60317	17	PC(P-37:1)	$[M+Na]^+$	PC	
810.6161	19	PC(38:4)	$[M+H]^+$	PC	[55]
812.62343	23	PC(38:3)	$[M+H]^+$	PC	[55]
814.55575a	20	PC(35:0)	$[M+K]^+$	PC	
820.54448	21	PC(36: 4)	$[M+Na]^+$	PC	[55]

826.59089	8	PC(P-37:0)	$[M+K]^+$	PC	
828.57068	18	PC(36:0)	$[M+K]^+$	PC	[55]
830.53114	33	PC(P-38:5)	$[M+K]^+$	PC	
832.60197	22	PC(38:4)	$[M+Na]^+$	PC	[55]
836.53845	20	PC(37:3)	$[M+K]^+$	PC	
844.54491	19	PC(38:6)	$[M+K]^+$	PC	[55]
846.55926	17	PC(38:5)	$[M+K]^+$	PC	[55]
856.60328	20	PC(38:0)[U]	$[M+K]^+$	PC	
872.57700	20	PC(40:6))	$[M+K]^+$	PC	[55]
896.50257	18	PC(41:1)	$[M+K]^+$	PC	
672.43388 [a]	22	PC(26:0)	$[M+Na]^+$	PC	
688.42946	20	PC(26:0)	$[M+K]^+$		
734.58424	10	PC(32:0)	$[M+H]^+$		[55]
772.54151	6	PC(32:0)	$M+K]^+$	PC	
798.55817	16	PC(34:1)	$M+K]^+$	PC	[55]
800.56994 [a]	20	PC(34:0)	$[M+K]^+$	PC	[55]
848.57717	21	PC(38:4)	$M+K]^+$	PC	[55]
852.51184	14	PI-Cer(t34:0(2OH))	$[M+K]^+$	Cer-PI	
351.15263	4	Ser Thr Ser Gly	$[M+H]^+$	Peptide	
383.14284	8	Pro Asp Gly Met	$[M+H-2H_2O]^+$	Peptide	
511.27947	5	Cys Tyr Cys Thr	$[M+Na]^+$	Peptide	
518.31127	2	Lys Asp Lys Lys	$[M+H]^+$	Peptide	
731.62011	5	TG(43:3)[iso3]	$[M+H]^+$	TG	
753.60379	8	TG(43:3)[iso6]	$[M+Na]^+$	TG	
769.57901	6	TG(39:3)[iso3]	$[M+K]^+$	TG	
932.58000	15	TH-Cer (d30:1)	$[M+H-2H_2O]^+$	TH-Cer	
199.00045	23	3-Phospho-D-erythronate	$[M+H-H_2O]^+$	Lipid fragments	
206.05814	1	N-Acetylphosphinothricin	[M+H-H2O]+	Lipid fragments	
222.03249	14	Phosphocholine	[M+K]+	Lipid fragments	
230.94900	10	2,3-Bisphospho-D-glycerate	$[M+H-2H_2O]^+$	Lipid fragments	
329.01119	20	D-glycero-D-manno-Heptose 1-phosphate	[M+K]+	Lipid fragments	
**9-AA assisted MALDI MS**					
716.09 [b]		PE(34:1)	$[M-H]^-$	PE	[60]
744.100 [b]		PE(36:1)	$[M-H]^-$	PE	[60]
746.96 [b]		PE(P-38:6)	$[M-H]^-$	PE	
762.89 [b]		PE(38:6)	$[M-H]^-$	PE	[60]
766.93 [b]		PE(38:4)	$[M-H]^-$	PE	[61]
794.80 b		PE(40:4)	[M-H]-	PE	
774.91 [b]		PS (p36:0)	$[M-H]^-$	PS	[60]
788.86 [b]		PS(36:1)	$[M-H]^-$	PS	[60]
834.75 b		PS(40:6)	[M-H]-	PS	[61]
806.81 [b]		ST(d18:1/18:0)	$[M-H]^-$	ST	[61]
822.76 [b]		ST(d18:1/18:0OH)	$[M-H]^-$	ST	[61]

850.72 [b]		ST(d18:1/h20:0OH)	$[M-H]^-$	ST	[60]
860.71 [b]		ST(d18:1/22:1)	$[M-H]^-$	ST	
862.73 [b]		ST(d18:1/22:0)	$[M-H]^-$	ST	[61]
874.68 [b]		ST(d18:1/22:2OH)	$[M-H]^-$	ST	
878.70 [b]		ST(d18:1/h22:0OH)	$[M-H]^-$	ST	[60]
888.70 [b]		ST(d18:1/24:1)	$[M-H]^-$	ST	[60,61]
904.60 [b]		ST(d18:1/24:1OH)	$[M-H]^-$	ST	[60]

[a] Molecules in the *m/z* column that had their precise molecular masses determined by FTICR MS and their fragmentation patterns acquired using MALDI-TOF/TOF MS/MS.

[b] Molecules in the *m/z* column that did not have their precise molecular masses determined by FTICR MS but their fragmentation patterns were acquired using MALDI-TOF/TOF MS/MS.

Molecules not marked in the *m/z* column had their precise molecular masses determined by FTICR MS. However, their fragmentation patterns were not acquired.

[c] Labeled references in the Ref. column report detection of the listed molecules in either rat or mouse brains with methods other than MALDI MS or LDI MS.

[d] Labeled references in the Ref. column describe listed molecules, but these molecules are not detected in the rodent brains.

References without labeling in the Ref. column describe detection of the listed molecules in either the rat or mouse brain with MALDI MS or LDI MS.

[e] The marked peptides in the Ref. column have structures of representative isomer of all the possible candidates for this precise *m/z* shown.

Abbreviations:

PC, Phosphatidylcholine; PE, Phosphatidylethanolamine; PA, Phosphatidic acid; CPA, cyclic phosphatidic acid; Cer-PE, Ceramide phosphoethanolamine; Cer-PI, Ceramide phosphoinositol; DAG, Diacylglycerol; TG, Triglyceride; Cer, Ceramide; GlcCer, Glucosylceramide; GalCer, Galactoceramide; THCer, Trihexosylceramide; M(IP)2C, Mannose-(inositol-P)2-ceramide; MIPC, Mannose-inositol-P-ceramide; FA, Fatty acid DHA, Docosahexaenoic acid; AA, Arachidonic acid; CL, Cholesterol; PS, Phosphatidylserine; ST, Sulfatide. **Amino Acids:** Ala, Alanine; Asn, Asparagine; Asp, Aspartate; Cys, Cysteine; Glu, Glutamate; Gln, Glutamine; Gly, Glycine; His, Histidine; Ile, Isoleucine; Leu, Leucine; Lys, Lysine; Met, Methionine; Phe, Phenylalanine; Pro, Proline; Ser, Serine; Thr, Threonine; Trp, Tryptophan; Tyr, Tyrosine; Val, Valine

Table S5. ANOVA results for data presented in Figure 5C and Figure S12B.

Hippocampal region	Factors	p values determined by ANOVA for different compounds					
		arecaidine	creatinine	cholesterol	DAG(34:1)	DAG(38:4)	PC(38:6)
CC	Interaction [a]	0.1	0.62	0.81	0.13	0.1	6.41E-04
	age [b]	0.02	0.002	0.37	0.01	0.005	7.04E-04
DG	interaction [a]	0.66	0.83	0.89	0.62	0.24	0.04
	age [b]	0.025	0.008	0.83	0.28	0.4	0.01
CA3	interaction [a]	0.36	0.1476	1.33E-04	1.39E-05	0.0026	0.0012
	age [b]	0.000428	3.33E-05	0.000122	3.77E-05	0.001	2.84E-06
CA1	interaction [a]	0.52	0.2	0.11	0.91	0.42	0.62
	age [b]	0.0061	0.0035	0.25	0.05	0.09	0.88

[a] Interaction is a change in the simple main effect of age over levels of paired comparison. If the value is <0.05, the interaction between age and paired comparison batch is significant.

[b] Effect of aging on the compound's average peak area. If the value is <0.05, the effect of aging is significant.

Abbreviations: CC, corpus callosum; DG, dentate gyrus; CA3, region III of hippocampus proper; CA1, region I of hippocampus proper.

References

1. J. A. Hankin, R. M. Barkley and R. C. Murphy, *J. Am. Soc. Mass Spectrom.*, 2007, **18**, 1646-1652.
2. E. J. Lanni, S. J. B. Dunham, P. Nemes, S. S. Rubakhin and J. V. Sweedler, *J. Am. Soc. Mass Spectrom.*, 2014, **25**, 1897-1907.
3. K. Shrivas, T. Hayasaka, Y. Sugiura and M. Setou, *Anal. Chem.*, 2011, **83**, 7283-7289.
4. E.-M. Ratai, L. Annamalai, T. Burdo, C.-G. Joo, J. P. Bombardier, R. Fell, R. Hakimelahi, J. He, M. R. Lentz, J. Campbell, E. Curran, E. F. Halpern, E. Masliah, S. V. Westmoreland, K. C. Williams and R. G. González, *Magn. Reson. Med.*, 2011, **66**, 625-634.
5. B. Shrestha, P. Nemes, J. Nazarian, Y. Hathout, E. P. Hoffman and A. Vertes, *Analyst*, 2010, **135**, 751-758.
6. M. Bisaglia, S. Mammi and L. Bubacco, *J. Biol. Chem.*, 2007, **282**, 15597-15605.
7. A. Napolitano, O. Crescenzi, A. Pezzella and G. Prota, *J. Med. Chem.*, 1995, **38**, 917-922.
8. S. Giri, J. R. Idle, C. Chen, T. M. Zabriskie, K. W. Krausz and F. J. Gonzalez, *Chem. Res. Toxicol.*, 2006, **19**, 818-827.
9. E. Rosengren, E. Lindereliasson and A. Carlsson, *J. Neural Transm.*, 1985, **63**, 247-253.
10. B. Fornstedt, I. Bergh, E. Rosengren and A. Carlsson, *J. Neurochem.*, 1990, **54**, 578-586.
11. J. R. Zgoda-Pols, S. Chowdhury, M. Wirth, M. V. Milburn, D. C. Alexander and K. B. Alton, *Toxicol. Appl. Pharmacol.*, 2011, **255**, 48-56.
12. R. D. Johnson, R. J. Lewis, D. V. Canfield and C. L. Blank, *J. Chromatogr. B Analyt. Technol. Biomed. Life Sci.*, 2004, **805**, 223-234.
13. P. Liu, N. Gupta, Y. Jing and H. Zhang, *Neuroscience*, 2008, **155**, 789-796.
14. D. S. Wishart, T. Jewison, A. C. Guo, M. Wilson, C. Knox, Y. Liu, Y. Djoumbou, R. Mandal, F. Aziat, E. Dong, S. Bouatra, I. Sinelnikov, D. Arndt, J. Xia, P. Liu, F. Yallou, T. Bjorndahl, R. Perez-Pineiro, R. Eisner, F. Allen, V. Neveu, R. Greiner and A. Scalbert, *Human Metabolome Database*, 2009, **3.0**.
15. M. Shariatgorji, A. Nilsson, R. J. A. Goodwin, P. Kallback, N. Schintu, X. Q. Zhang, A. R. Crossman, E. Bezard, P. Svenningsson and P. E. Andren, *Neuron*, 2014, **84**, 697-707.
16. C. Esteve, E. A. Tolner, R. Shyti, A. van den Maagdenberg and L. A. McDonnell, *Metabolomics*, 2016, **12**.
17. Y. Xing, X. Li, X. Guo and Y. Cui, *Anal. Bioanal. Chem.*, 2016, **408**, 141-150.
18. A. F. Steulet, H. J. Mobius, S. J. Mickel, K. Stocklin and P. C. Waldmeier, *Biochem. Pharmacol.*, 1996, **51**, 613-619.
19. S. Kaul, M. D. Faiman and C. E. Lunte, *Electrophoresis*, 2011, **32**, 284-291.
20. C. D. Schmoutz, G. F. Guerin, S. P. Runyon, S. Dhungana and N. E. Goeders, *Behav. Brain Res.*, 2015, **291**, 108-111.
21. K. Inoue, Y. Miyazaki, K. Unno, J. Z. Min, K. Todoroki and T. Toyo'oka, *Biomed. Chromatogr.*, 2016, **30**, 55-61.
22. H. L. Wang, K. Q. Lian, B. Han, Y. Y. Wang, S. H. Kuo, Y. Geng, J. Qiang, M. Y. Sun and M. W. Wang, *J. Alzheimers Dis.*, 2014, **39**, 841-848.
23. S. Kolbaev and A. Draguhn, *Eur. J. Neurosci.*, 2008, **28**, 535-545.
24. A. Ichiyama, S. Nakamura, H. Kawai, T. Honjo, Nishizuk.Y, O. Hayaishi and S. Senoh, *J. Biol. Chem.*, 1965, **240**, 740-749.
25. C. Fred Hassman, J. T. Pelton, S. H. Buck, P. Shea, E. F. Heminger, R. J. Broersma and J. M. Berman, *Biochem. Biophys. Res. Commun.*, 1988, **152**, 1070-1075.
26. E. E. Jones, S. Dworski, D. Canals, J. Casas, G. Fabrias, D. Schoenling, T. Levade, C. Denlinger, Y. A. Hannun, J. A. Medin and R. R. Drake, *Anal. Chem.*, 2014, **86**, 8303-8311.
27. S. Theofilopoulos, W. J. Griffiths, P. J. Crick, S. Z. Yang, A. Meljon, M. Ogundare, S. S. Kitambi, A. Lockhart, K. Tuschl, P. T. Clayton, A. A. Morris, A. Martinez, M. A. Reddy, A. Martinuzzi, M. T. Bassi, A. Honda, T. Mizuochi, A. Kimura, H. Nittono, G. De Michele, R.

Carbone, C. Criscuolo, J. L. Yau, J. R. Seckl, R. Schule, L. Schols, A. W. Sailer, J. Kuhle, M. J. Fraidakis, J. A. Gustafsson, K. R. Steffensen, I. Bjorkhem, P. Ernfors, J. Sjovall, E. Arenas and Y. Q. Wang, *J. Clin. Invest.*, 2014, **124**, 4829-4842.
28. J. I. Jung, A. R. Price, T. B. Ladd, Y. Ran, H. J. Park, C. Ceballos-Diaz, L. A. Smithson, G. Hochhaus, Y. F. Tang, R. Akula, S. Ba, E. H. Koo, G. Shapiro, K. M. Felsenstein and T. E. Golde, *Mol. Neurodegener.*, 2015, **10**, 29.
29. L. B. Xu, M. Kliman, J. G. Forsythe, Z. Korade, A. B. Hmelo, N. A. Porter and J. A. McLean, *J. Am. Soc. Mass Spectrom.*, 2015, **26**, 924-933.
30. S. Vascellari, S. Banni, C. Vacca, V. Vetrugno, F. Cardone, M. A. Di Bari, P. La Colla and A. Pani, *Lipids Health Dis.*, 2011, **10**, 132.
31. N. E. Manicke, M. Nefliu, C. P. Wu, J. W. Woods, V. Reiser, R. C. Hendrickson and R. G. Cooks, *Anal. Chem.*, 2009, **81**, 8702-8707.
32. A. N. Lazar, C. Bich, M. Panchal, N. Desbenoit, V. W. Petit, D. Touboul, L. Dauphinot, C. Marquer, O. Laprevote, A. Brunelle and C. Duyckaerts, *Acta Neuropathol.*, 2013, **125**, 133-144.
33. S. Sole-Domenech, P. Sjovall, V. Vukojevic, R. Fernando, A. Codita, S. Salve, N. Bogdanovic, A. H. Mohammed, P. Hammarstrom, K. P. R. Nilsson, F. M. LaFerla, S. Jacob, P. O. Berggren, L. Gimenez-Llort, M. Schalling, L. Terenius and B. Johansson, *Acta Neuropathol.*, 2013, **125**, 145-157.
34. M. Dufresne, A. Thomas, J. Breault-Turcot, J. F. Masson and P. Chaurand, *Anal. Chem.*, 2013, **85**, 3318-3324.
35. C. Bich, D. Touboul and A. Brunelle, *Int. J. Mass Spectrom.*, 2013, **337**, 43-49.
36. M. Lorenz, O. S. Ovchinnikova, V. Kertesz and G. J. Van Berkel, *Rapid Commun. Mass Spectrom.*, 2013, **27**, 1429-1436.
37. K. Yang, C. M. Jenkins, B. Dilthey and R. W. Gross, *Anal. Bioanal. Chem.*, 2015, **407**, 5199-5210.
38. L. Muller, A. Kailas, S. N. Jackson, A. Roux, D. C. Barbacci, J. A. Schultz, C. D. Balaban and A. S. Woods, *Kidney Int.*, 2015, **88**, 186-192.
39. S. Bobba, G. E. Resch and W. G. Gutheil, *Anal. Biochem.*, 2012, **425**, 145-150.
40. S. Himmelseher, E. Pfenninger and P. Herrmann, *J. Parenter. Enteral. Nutr.*, 1996, **20**, 281-286.
41. M. Zollinger, U. Amsler and J. Brauchli, *J. Chromatogr.*, 1990, **532**, 27-36.
42. K. R. Amaya, J. V. Sweedler and D. F. Clayton, *J. Neurochem.*, 2011, **118**, 499-511.
43. S. M. Lam, L. Tong, B. Reux, M. J. Lear, M. R. Wenk and G. Shui, *J. Chromatogr.*, 2013, **1308**, 166-171.
44. G. Barcelo-Coblijn, M. L. Martin, C. Egea, M. Y. Golovko, E. J. Murphy and P. V. Escriba, *Chem. Phys. Lipids*, 2008, **154**, S43-S44.
45. T. Hayasaka, N. Goto-Inoue, N. Zaima, K. Shrivas, Y. Kashiwagi, M. Yamamoto, M. Nakamoto and M. Setou, *J. Am. Soc. Mass Spectrom.*, 2010, **21**, 1446-1454.
46. Y. H. Lin and N. Salem, *J. Lipid Res.*, 2005, **46**, 1962-1973.
47. N. Hussein, I. Fedorova, T. Moriguchi, K. Hamazaki, H.-Y. Kim, J. Hoshiba and N. Salem, Jr., *Lipids*, 2009, **44**, 685-702.
48. M. Igarashi, K. Ma, F. Gao, H.-W. Kim, S. I. Rapoport and J. S. Rao, *J. Alzheimers Dis.*, 2011, **24**, 507-517.
49. T. B. Angerer, M. D. Pour, P. Malmberg and J. S. Fletcher, *Anal. Chem.*, 2015, **87**, 4305-4313.
50. G. B. Wells, R. C. Dickson and R. L. Lester, *J. Biol. Chem.*, 1998, **273**, 7235-7243.
51. P. A. Sutton and D. A. Buckingham, *Acc. Chem. Res.*, 1987, **20**, 357-364.
52. S. L. Morales-Lazaro, B. Serrano-Flores, I. Llorente, E. Hernandez-Garcia, R. Gonzalez-Ramirez, S. Banerjee, D. Miller, V. Gududuru, J. Fells, D. Norman, G. Tigyi, D. Escalante-Alcalde and T. Rosenbaum, *J. Biol. Chem.*, 2014, **289**, 24079-24090.
53. P. Sjövall, B. Johansson and J. Lausmaa, *Appl. Surf. Sci.*, 2006, **252**, 6966-6974.
54. H. Kettling, S. Vens-Cappell, J. Soltwisch, A. Pirkl, J. Haier, J. Müthing and K. Dreisewerd, *Anal. Chem.*, 2014, **86**, 7798-7805.

55. J. A. Fernández, B. Ochoa, O. Fresnedo, M. T. Giralt and R. Rodríguez-Puertas, *Anal. Bioanal. Chem.*, 2011, **401**, 29-51.
56. R. Weng, S. Shen, L. Yang, M. Li, Y. Tian, Y. Bai and H. Liu, *Rapid Commun. Mass Spectrom.*, 2015, **29**, 1491-1500.
57. T. Houjou, K. Yamatani, M. Imagawa, T. Shimizu and R. Taguchi, *Rapid Commun. Mass Spectrom.*, 2005, **19**, 654-666.
58. E. J. Ahn, H. Kim, B. C. Chung, G. Kong and M. H. Moon, *J. Chromatogr.*, 2008, **1194**, 96-102.
59. S. N. Jackson, H.-Y. J. Wang and A. S. Woods, *Anal. Chem.*, 2005, **77**, 4523-4527.
60. C. D. Cerruti, F. Benabdellah, O. Laprevote, D. Touboul and A. Brunelle, *Anal. Chem.*, 2012, **84**, 2164-2171.
61. A. Thomas, J. L. Charbonneau, E. Fournaise and P. Chaurand, *Anal. Chem.*, 2012, **84**, 2048-2054.
